# Continuous and discontinuous compressible flows in a converging–diverging channel solved by physics-informed neural networks without exogenous data

**DOI:** 10.1038/s41598-024-53680-2

**Published:** 2024-02-15

**Authors:** Hong Liang, Zilong Song, Chong Zhao, Xin Bian

**Affiliations:** 1 Department of Physics, Hangzhou Dianzi University, Hangzhou, 310018 China; 2 Hangzhou Shiguangji Intelligient Electronics Technology Co., Ltd, Hangzhou, 310018 China; 3 State Key Laboratory of Fluid Power and Mechatronic Systems, Department of Engineering Mechanics, Zhejiang University, Hangzhou, 310027 China

**Keywords:** Unsteady compressible flow, Normal shock, Physics-informed neural networks, Direct numerical simulation, Aerospace engineering, Computational science

## Abstract

Physics-informed neural networks (PINNs) are employed to solve the classical compressible flow problem in a converging–diverging nozzle. This problem represents a typical example described by the Euler equations, a thorough understanding of which serves as a guide for solving more general compressible flows. Given a geometry of the channel, analytical solutions for the steady states do indeed exist, and they depend on the ratio between the back pressure of the outlet and the stagnation pressure of the inlet. Moreover, in the diverging region, the solution may branch into subsonic flow, supersonic flow, or a mixture of both with a discontinuous transition where a normal shock occurs. Classical numerical schemes with shock fitting and capturing methods have been developed to solve this type of problem effectively, whereas the original PINNs are unable to predict the flows correctly. We make a first attempt to exploit the power of PINNs to solve this problem directly by adjusting the weights of different components of the loss function to acquire physical solutions and in the meantime, avoid trivial solutions. With a universal setting yet no exogenous data, we are able to solve this problem accurately; that is, for different given pressure ratios, PINNs provide different branches of solutions at both steady and unsteady states, some of which are discontinuous in nature. For an inverse problem such as unknown specific-heat ratio, it works effectively as well.

## Introduction

Euler equations embrace the conservation laws for inviscid fluids, which are often compressible at high speed^1^. It is infeasible to derive an analytical solution for this type of equations except in a few special cases. A discontinuous solution associated with shock wave may be generated due to the hyperbolic and non-linear properties of the partial differential equations (PDEs) when the fluid moves at speeds comparable to its speed of sound^2^. This further poses challenges for the development of numerical schemes and therefore, have induced many ingenious efforts in the past a few decades^3,4^. There is generally a trade-off between accuracy and stability in the numerical methods^5^. The low order methods can produce stable but less accurate results, where sharp profiles of shocks are smoothed. To the contrary, the high-order methods are able to generate relatively accurate results, but are often troubled by instabilities and Gibbs phenomenon near the discontinuities^6^. Furthermore, the requirement of stability also imposes a strict CFL limit to the numerical methods, resulting in small time steps in simulations. These facts render the simulations by traditional numerical methods both human-intelligence concentrated and computationally intensive^7–10^.

Recently, machine learning methods, in particular, the deep neural networks (DNNs) have received enormous attentions and are trying to replace human pounding by network training and inferring. Due to the tremendous successes in other fields, they also quickly became an alternative approach to solve PDEs^11–14^. When solving the Euler equations, DNNs can be embedded into the traditional numerical methods to facilitate accurate results^15–17^. As another paradigm driven by the date science, DNNs can be trained by a large amount of analytical/experimental/simulation data, corresponding to the so-called *supervised learning*. Once trained, the DNNs can offer solutions for the PDEs in interpolated and even slightly extrapolated space of parameters much faster than the traditional numerical schemes. However, data are not always abundant in realistic applications or only partially accessible at best. To address this issue, physics-informed neural networks (PINNs) are proposed and trained by combining physics laws in the form of PDEs together with available data^18^. In contrast to the undecorated DNNs, PINNs can be trained with data in shortage or even missing to solve a forward problem described by known PDEs. This corresponds to an *unsupervised or weakly supervised learning*. Moreover, PINNs can also deal with an inverse problem at ease, where some coefficient values in the PDEs are unclear, such as the viscosity in the Navier-Stokes equations^19^. Since its invention, there have already been many innovative works to improve the accuracy and efficiency of PINNs^20–25^. Concerning the discontinuous solutions of PDEs, quite a few work have been conducted with PINNs. In the seminal work of PINNs^18^, a viscous term is added to smooth the shock produced by the Burgers’ equation. Mao et al.^26^ choose to distribute more sampling points in the discontinuous region identified beforehand, forming a cluster of points for a better training. Compared with uniformly or randomly sampled distribution of points, their strategy achieves a higher precision. The conservative PINNs proposed by Jagtap et al.^27^ solve the continuous and discontinuous regions separately, where in the discontinuous part a larger network and more data are selected for training. Their work demonstrates that a special division of the training area can get more accurate solutions than that of other conventional divisions. Moreover, Patel et al. propose control-volume based PINNs^28^ and combine them with a finite volume method, when no derivative exists in the discontinuous part. They define a loss function for a single control volume and obliterate derivative operation by integrating the equation, to ensure a correct solution. It is worth mentioning that the Boltzmann equation with the Bhatnagar-Gross-Krook model has been incorporated into PINNs so that both continuum and rarefied gas flows can be resolved ^29,30^.

Different from previous works, we aim to employ PINNs to solve a forward problem of compressible flows *without exogenous data*. The input data for the boundary and initial conditions of the PDEs are not part of the solution and not considered as training/labeled data. Therefore, this corresponds to an unsupervised learning. In addition, we also apply PINNs to solve a representative inverse problem such as the unknown specific-heat ratio, where abundant data are provided in the solution domain. This corresponds to a supervised learning. In particular, we are interested in a typical flow problem taking place in a so-called Laval nozzle or converging–diverging (CD) nozzle. Its actual solution depends on the ratio between the pressures at the outlet and inlet, and in the diverging region it may branch into subsonic flow, supersonic flow, and a mixture of both with discontinuous transition where a normal shock takes place. Since both analytical and numerical solutions for steady states exist in textbooks^1,3^, this problem serves as an ideal benchmark to examine PINNs’ performance for continuous and discontinuous compressible flows. A thorough understanding of the procedure for seeking solutions of this simple problem may provide a guidance to solve more general compressible flows, whether there is auxiliary data or not.

**Figure 1:**
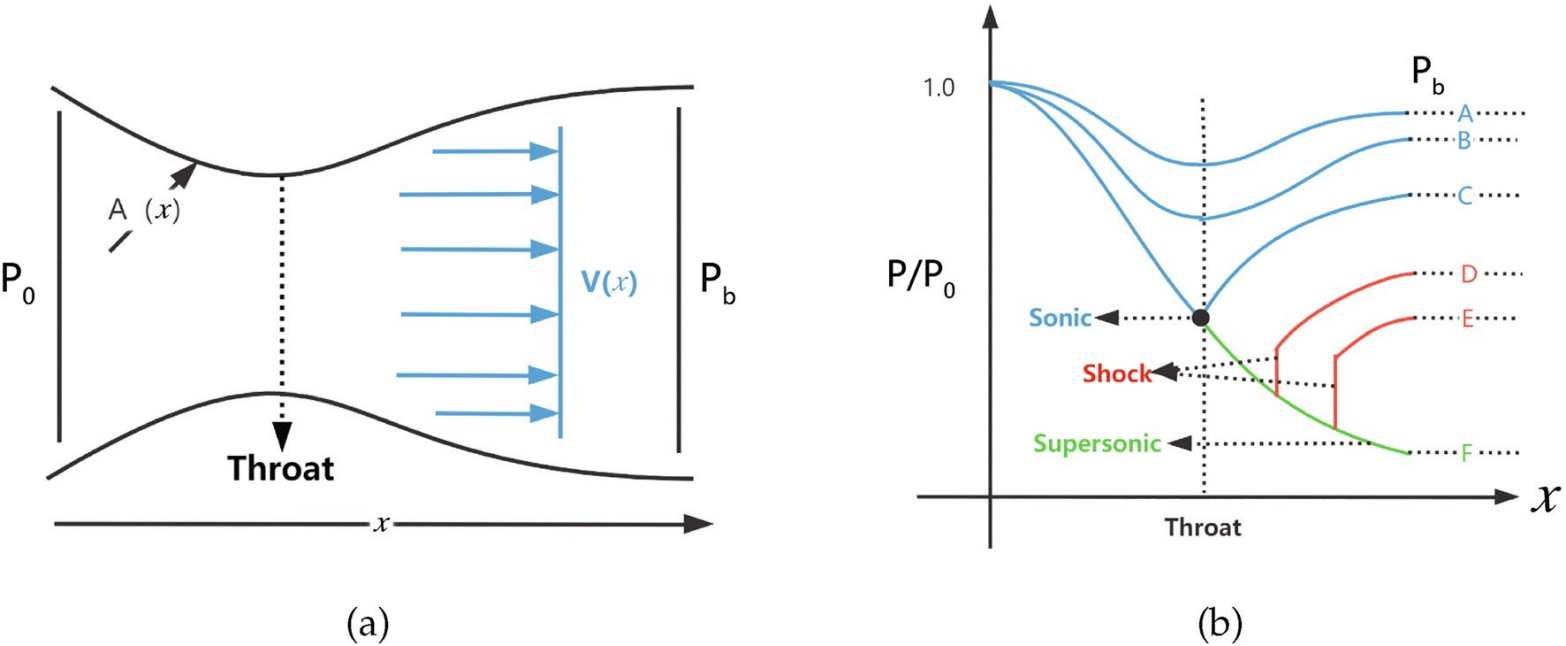
(**a**) one dimensional approximation to the flow within a converging–diverging (CD) nozzle; (**b**) pressure distributions and flow characteristics along the CD nozzle according to various ratios between the back/outlet pressure and stagnation/inlet pressure. Curves A and B correspond to subsonic flows; curve F corresponds to a subsonic-supersonic flow; curve C corresponds to the critical subsonic flow, where the flow is subsonic all over except that at the throat it reaches exactly the speed of sound; curve D and E correspond to flows with norm shocks in-between supersonic-subsonic transitions.

A typical three-dimensional CD nozzle with axis-symmetry is shown in Fig. 1a. We assume that the velocity has only one component in the *x* direction *V*(*x*) and it changes with the area *A*(*x*) of the cross section, which varies smoothly and slowly. Therefore, one-dimensional equations are adequate to describe the flows. The smallest area named as throat controls the flow rate when the flow is supersonic in the diverging part. According to the pressure ratio between the back pressure $P_{b}$ at the outlet and stagnation pressure $P_0$ at the inlet, flow characteristics vary, as shown in Fig. 1b. In particular, flows with a mixture of subsonic-supersonic-subsonic properties take place, as curves D or E, when $P_b$ is moderate. Normal shock waves are generated for the transition from supersonic back to subsonic flows to raise the inner pressure up to conform with the back pressure at the outlet. The generation of shock wave is a non-isentropic process and manifests itself as a discontinuity in the context of continuum mechanics, which renders reliable solutions of the traditional numerical methods difficult.

In this work, we adopt a specific version of PINNs with a universal setting to resolve the flows in the CD nozzle with only initial and boundary conditions, whether there is a shock wave or not, without any auxiliary data. By varying $P_b$, it offers different branches of solutions correctly. If there is a normal shock, it identifies the shock location accurately and meanwhile provides a sharp solution. This is in contrast to the previous work ^26^, where the discontinuous profile is given already as the initial condition. The structure of the paper is arranged as follows. In "The method" section, flow equations together with the structure and parameters of PINNs are introduced. In "Steady state solutions" section, hard constraints on the boundaries are introduced and weights of different loss functions are universally determined so that different flow characteristics are predicted at steady states. One inverse problem with unknown specific-heat ratio for the discontinuous flow is also demonstrated. In "Time-dependent solutions" section, unsteady flows are solved and the influence of different hyper-parameters of the neural networks is analyzed to successfully improve the accuracy of solutions. In "Solutions in conservative form" section, governing equations in conservative form are discussed and finally, In "Conclusions" section a summary is made.

## The method

The one-dimensional Euler’s equations are adopted to describe the flows and more specifically, a simple differential form is initially considered

1 $$\begin{aligned} \left\{ \begin{aligned}{}&A\frac{\partial \rho }{\partial t}+Av\frac{\partial \rho }{\partial x} +A\rho \frac{\partial v }{\partial x}+v\rho \frac{\partial A }{\partial x}=0,\\&A\rho \frac{\partial v}{\partial t}+Av\rho \frac{\partial v }{\partial x}+A\frac{\partial P}{\partial x} =0,\\&A\rho \frac{\partial T}{\partial t}+Av\rho \frac{\partial T }{\partial x}+P\left( A\frac{\partial v}{\partial x}+v\frac{\partial A}{\partial x} \right) =0 ,\\&P-\rho RT =0. \end{aligned} \right. \end{aligned}$$

Here $\rho$, *v*, *T*, and *P* are density, velocity, temperature, and pressure, respectively. *R* is the universal gas constant. We fix the stagnation properties such as density $\rho _0$, temperature $T_0$, and pressure $P_0$ at the inlet (left) and adjust the the back pressure $P_{b}$ at the outlet (right), to generate different flow regimes in the CD nozzle, as shown in Fig. 1. A typical set of stagnation values are $\rho _0 = 1.52 kg/m^{3}$, $T_0 = 286.1 K$, and $P_0 = 1.247\times 10^{5} N/m^{2}$, respectively.

Flow properties are made dimensionless by $\rho _0$, $T_0$ and throat area $A^*$ as follows:

$$\begin{aligned} \rho ' = \frac{\rho }{\rho _{0} }, T' = \frac{T}{T_{0} }, A'=\frac{A}{A^{*}}. \end{aligned}$$

Therefore, the sound speed at stagnation is $a_{0}=\sqrt{\gamma RT_0}$, where $\gamma =1.4$ is the specific-heat ratio of air. Furthermore, $P' = P/P_{0}$, $v'=v/a_{0}$, and $x'=x/\sqrt{A^*}$, $t'=ta_{0}/\sqrt{A^*}$. Eq. (1) become dimensionless as

2 $$\begin{aligned} \left\{ \begin{aligned}{}&A'\frac{\partial \rho ' }{\partial t' } +A' v' \frac{\partial \rho ' }{\partial x' } +A' \rho ' \frac{\partial v' }{\partial x' }+v\rho \frac{\partial A' }{\partial x' }=0,\\&A'\left( \gamma \rho ' \frac{\partial v' }{\partial t' }+\gamma v' \rho ' \frac{\partial v' }{\partial x' }+\frac{\partial P' }{\partial x' }\right) =0,\\&A' \rho ' \frac{\partial T' }{\partial t' }\left( \frac{1}{\gamma -1 }\right) +A' v' \rho ' \frac{\partial T' }{\partial x' }\left( \frac{1}{\gamma -1 }\right) +P' \left( A' \frac{\partial v' }{\partial x' }+v\frac{\partial A' }{\partial x' } \right) =0, \\&P'-\rho ' T{'} = 0. \end{aligned} \right. \end{aligned}$$

In the rest of this paper, all physical quantities shall appear in dimensionless form. Therefore, we remove all “ $'$ ”s in the equations for convenience.

**Figure 2:**
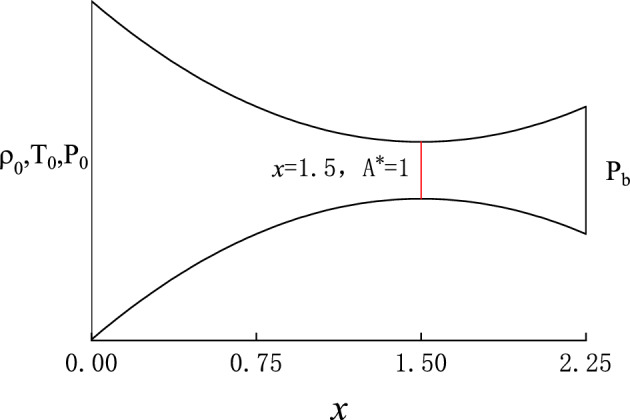
A converging–diverging nozzle with cross-section area *A* described by a parabolic function of *x* along the axis with $x\in (0,2.25)$, where the throat has the minimum area $A^*=1$ at $x=1.5$. $\rho _{0}$, $T_{0}$ and $P_{0}$ are the stagnation density, temperature, and pressure at the inlet (left), respectively. $P_{b}$ is the back pressure at the outlet (right). We adjust $P_{b}$ to vary the pressure ratio and to generate different flow regimes in the nozzle.

We define the cross-section of the CD nozzle as a parabolic function along the *x* axis: $A(x)=1+2.2*(x-1.5)^{2}$, where the minimum area $A^*=1$ takes place at $x=1.5$, as shown in Fig. 2. This geometry is nothing special and only taken for convenience. The methodology presented later also applies to more general geometries.

**Figure 3:**
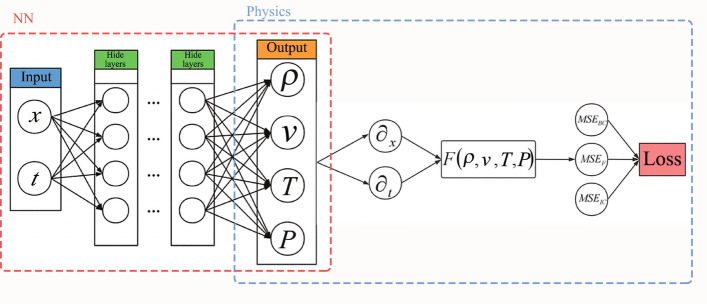
The structure of PINNs. On the left is a simple feedforward neural network to be trained while on the right is the physics information expressed in PDEs. A loss function composed of boundary conditions, initial conditions, and physics equations together guides the training of the neural network.

The structure of the neural networks (NNs) is presented in Fig. 3, where it is trained without any auxiliary data, except for the boundary and initial conditions. The loss function of the NNs is expressed as follows

3 $$\begin{aligned} Loss=Loss_{BC}+Loss_{IC}+Loss_{F}, \end{aligned}$$

where $Loss_{BC}$, $Loss_{IC}$, and $Loss_{F}$ correspond to the sub-loss function of boundary conditions, initial conditions, and PDEs, respectively. Note that $Loss_{IC}$ is activated only when the unsteady solution is required for the flow. This is also true for time *t* as input for the neural networks. The sub-loss function of the PDEs has four components originated from the four equations in Eq. (2)

4 $$\begin{aligned} \left\{ \begin{aligned}{}&A\frac{\partial \rho }{\partial t } +A v \frac{\partial \rho }{\partial x } +A \rho \frac{\partial v }{\partial x }+v\rho \frac{\partial A }{\partial x }=F_1(x,t),\\&A\left( \gamma \rho \frac{\partial v }{\partial t }+\gamma v \rho \frac{\partial v }{\partial x }+\frac{\partial P }{\partial x }\right) =F_2(x,t),\\&A \rho \frac{\partial T }{\partial t }\left( \frac{1}{\gamma -1 }\right) +A v \rho \frac{\partial T }{\partial x }\left( \frac{1}{\gamma -1 }\right) +P \left( A \frac{\partial v }{\partial x }+v\frac{\partial A }{\partial x } \right) =F_3(x,t), \\&P-\rho T = F_4(x,t). \end{aligned} \right. \end{aligned}$$

Here $F_1(x,t)$, $F_2(x,t)$, $F_3(x,t)$, and $F_4(x,t)$ represent residuals of the mass, momentum, energy, and state equations, respectively. We assume that pressure *P* and density $\rho$ are two individual variables so that the NNs have both variables as outputs. Accordingly, *P* and $\rho$ form a sub-loss function $F_4(x, t)$ via the residual from the equation of state. Each component of the loss function has an associated weight $\omega _i$ and in part they determine the optimization of the network parameters:

5 $$\begin{aligned} F=\omega _1 F_1+ \omega _2 F_2+ \omega _3 F_3+ \omega _4 F_4. \end{aligned}$$

By default $w_i=1$ for $i=1,2,3,4$. All the sub-loss functions are expressed in the form of mean squared errors (MSEs)

6 $$\begin{aligned} Loss_{BC}&= MSE_{BC}, 
\end{aligned}$$

7 $$\begin{aligned} Loss_{IC}&= MSE_{IC}, \end{aligned}$$

8 $$\begin{aligned} Loss_{F}&= MSE_{F} = \sum _{i=1}^{4} \frac{\omega _i}{N_{F} } \sum _{j = 1}^{N_{F}} \left| F_{i}\left( x_{j} ,t_{j} \right) \right| ^{2}, \end{aligned}$$

where $F_i\left( x_{j},t_{j} \right)$ is the corresponding residual at point $(x_j, t_j)$ among all $N_F$ training points. The actual expressions of $Loss_{BC}$ and $Loss_{IC}$ are problem-dependent and shall be described in later sections. Because the NNs adopt the chain rule of derivatives, we do not need to approximate the partial differential terms by any special numerical scheme as in the traditional numerical methods. In fully connected neural networks, the relationship between the output and input can be described by explicit mathematical expressions and thus, the partial derivatives of the output with respect to the input can be simply expressed. Therefore, each residual $F_i$ can be expressed steadily. In the process of minimizing the loss function towards zero, the back propagation algorithm optimizes the parameters (weights and biases) of the NNs.^31^. In this context, the optimization process is also named as training. Once trained, the NNs can predict values $\rho$, *v*, *T*, and *P* for any given *x* and *t*.

## Steady state solutions

We commence to solve for flow problems within the CD nozzle at steady states, therefore the terms containing partial derivative with respect to time in Eq. (2) and (4) are temporarily discarded. For the steady states, we can obtain accurate solutions via analytical methods and make use of them to evaluate the performance of PINNs. In "Diverging channel" section we solve the flows in the diverging part of the nozzle by imposing the critical states of the throat as boundary conditions. In "Converging-diverging nozzle" section, we capture the shock wave by modifying the NNs and obtain accurate solutions at a high resolution of sampling points. In "Effects of resolution" section, we investigate the effects due to the number of training points on the accuracy of solutions. In this section, the input to the neural network is only *x*. The parameter settings of the neural network used in each subsection are summarized in Table 1 and will be elaborated in the text.

**Table 1:** List of NNs’ parameters for steady state solutions. The size of the neural network is uniformly 3 hidden layers, each layer has 30 neurons. In "Effects of resolution" section, we examine various numbers of training points.

Subsection	Parameters			
	BC points	Training points	Adam epochs	LBFGS epochs
3.1	1	200	2000	500
3.2	2	2000	20000	5000
3.3	2	N.A.	20000	5000
3.4	2	2000	25000	5000

### Diverging channel

The flow characteristics are relatively simple in the converging- part of the CD nozzle, whereas it is rather complex in the diverging part. Therefore, we first impose the physical quantities at the throat as inlet boundary conditions and calculate flows in the diverging part alone. The analytical solutions to this problem are expressed as follows,

9 $$\begin{aligned} \left\{ \begin{aligned}{}&\left[ {1+\frac{1}{2} \left( \gamma -1 \right) Ma^{2} } \right] ^{\frac{1 }{\left( \gamma -1 \right) } }= \frac{1}{\rho },\\&\frac{1}{Ma} \left[ \frac{1+\frac{1}{2} \left( \gamma -1 \right) Ma^{2} }{\frac{1}{2} \left( \gamma +1 \right) } \right] ^{\frac{1}{2}\left( \gamma +1 \right) \left( \gamma -1 \right) }=\frac{1}{A^* },\\&{1+\frac{1}{2} \left( \gamma -1 \right) Ma^{2} }=\frac{1}{T },\\&\left[ {1+\frac{1}{2} \left( \gamma -1 \right) Ma^{2} } \right] ^{\frac{\gamma }{\left( \gamma -1 \right) } }=\frac{1}{P }, \end{aligned} \right. \end{aligned}$$

which are valid for both subsonic and supersonic flows. As they are not in explicit form, some iterative procedures are necessary and we adopt a web-based applet to calculate accurate solutions with 8 decimal digits as references^32^.

More specifically, we take the critical state of air reaching the speed of sound at the throat. Accordingly, the inlet boundary conditions for the diverging channel are

$$\begin{aligned} (\rho _{x=1.5},v_{x=1.5},T_{x=1.5},P_{x=1.5})=(0.634, 0.912, 0.833, 0.528). \end{aligned}$$

Given the geometry, analytical solution may be subsonic, supersonic and a mixture of both with a discontinuous shock. Both subsonic and supersonic solutions are smooth and unique, such as *C* and *F* curves on Fig. 1b, and are available from Eq. (9). Moreover, there are infinitely many solutions, each of which has a discontinuous shock, such as *D* and *E* curves on Fig. 1b and more are not shown. Each solution is unique corresponding to one specific boundary condition at the outlet. Out of curiosity, however, we intentionally leave the outlet boundary for free, to interrogate what PINNs generate.

The computational domain is for $x\in \left[ 1.5,2.25 \right]$ and the loss function expressed as MSEs for the inlet boundary is defined as

10 $$\begin{aligned} MSE_{BC} =MSE_{\rho }+MSE_{v}+MSE_{T}+MSE_{P}, \end{aligned}$$

with:

11 $$\begin{aligned} \begin{aligned}{}&MSE_{\rho } = \frac{1}{N_{BC} } \sum _{j = 1}^{N_{BC}} \left( \left| \rho _{NN}\left( x_{j}=1.5 \right) -\rho (x_{j}=1.5)\right| ^{2}\right) ,\\&MSE_{v} = \frac{1}{N_{BC} } \sum _{j = 1}^{N_{BC}} \left( \left| v_{NN}\left( x_{j}=1.5 \right) -v(x_{j}=1.5)\right| ^{2}\right) ,\\&MSE_{T} = \frac{1}{N_{BC} } \sum _{j = 1}^{N_{BC}} \left( \left| T_{NN}\left( x_{j}=1.5 \right) -T(x_{j}=1.5)\right| ^{2}\right) ,\\&MSE_{P} = \frac{1}{N_{BC} } \sum _{j = 1}^{N_{BC}} \left( \left| P_{NN}\left( x_{j}=1.5 \right) -P(x_{j}=1.5)\right| ^{2}\right) . \end{aligned} \end{aligned}$$

Here the values with subscript “$_{NN}$” are the ones predicted by the NN, while the bare values are imposed ones. NNs’ parameters for weight and bias are initialized by Glorot scheme^33^. This convention also applies later on. The NN has 3 hidden layers, each layer has 30 neurons, and tanh is the activation function. We choose $N_{BC}=1$ and $N_F=100$ points in the x direction, for the inlet boundary and collocation points within the physical domain, respectively. To investigate clearly the influence of other parameters on the results, we intentionally choose all training points uniformly distributed.

**Figure 4:**
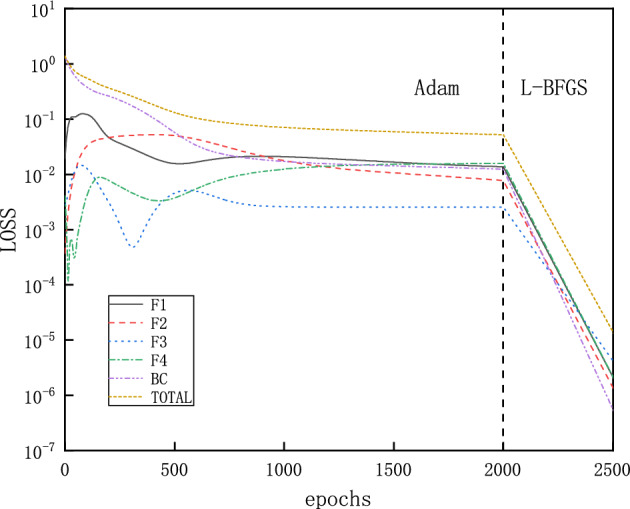
Evolution of the loss function for a typical instance of PINNs for solving the flows in the diverging channel: with Adam and L-BFGS optimizers, each component of the loss function descends towards minimization with more training epochs.

The training proceeds with Adam optimizer of learning rate 0.0001 for 2000 epochs and continues with L-BFGS optimizer for 500 epochs^34,35^. The Adam optimizer can quickly find the vicinity of the optimal solution in the early stage of training, while the L-BFGS can further optimize the parameters and improve the accuracy of the model in the subsequent training. Initial training with Adam optimizer and then training with L-BFGS can take full advantage of these two optimization algorithms to improve the convergence speed and accuracy of the model. For problems of varying complexity, different numbers of epochs are used by trial and error. A typical evolution of the loss function is shown in Fig. 4, where each component descends clearly towards minimization with more training epochs. After completion of the training, 50 evenly distributed points in the computational domain are selected for predicting $\rho$, $Ma=v/c=v/\sqrt{\gamma R T}$, *T* and *P*, which corresponds to a simple forward pass of the NNs. Since both sets of points are evenly distributed, the prediction points are actually a subset of the training points. This convention also applies later on, unless otherwise stated.

**Figure 5:**
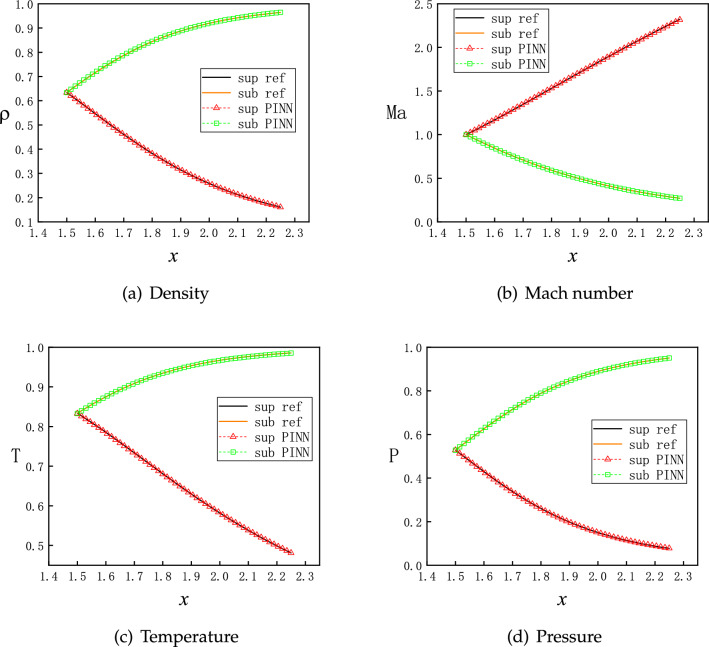
Solutions for flows in the diverging channel. Only inlet boundary conditions are imposed while the outlet boundary conditions are intentionally left free. Each running instance of PINNs generates an accurate supersonic or subsonic solution randomly, due to the inherent randomness during the initialization of the NN’s parameters. Furthermore, PINNs deliberately avoid the subtle branch of infinite many solutions, which involve discontinuous shocks.

With the same setting, we run multiple instances of PINNs to predict the flow solutions. Surprisingly, for each instance we may obtain one of two different solutions randomly. One is for supersonic flows and the other is for subsonic flows, as shown in Fig. 5. Moreover, both the subsonic and supersonic solutions of PINNs are in excellent agreement with the analytical ones, described by Eq. (9). After many instances of training and prediction, this observation is repeatable, that is, without providing the outlet boundary condition, PINNs are always able to find one of the two smooth solutions. We attribute this uncertain outcome to the random initialization of the NN’s parameters^33^. Nevertheless, without an appropriate boundary condition for the outlet, PINNs deliberately circumvent the discontinuous solutions. This setup is not well-defined mathematically, as the outlet boundary condition is left free, there are potentially infinite numbers of solutions including discontinuous ones. We speculate that the local minima for discontinuous solutions on the landscape of the loss function are relatively much higher, and therefore we obtain only two continuous solutions.

### Converging–diverging nozzle

We continue to consider the whole geometry of the CD nozzle. Stagnation values of density, temperature, and pressure are given at the inlet, while a back pressure $P_b$ is provided at the outlet. Depending on the value of $P_b$, different flow characteristics occur. For smooth solutions of both subsonic and supersonic flows, the analytical expressions in Eq. (9) are utilized. For a solution with a normal shock in the diverging part, we refer to the Rankine–Hugoniot equations as follows:

12 $$\begin{aligned} \left\{ \begin{aligned}{}&\frac{(\gamma +1)Ma_{1}^{2} }{(\gamma -1)Ma_{1}^{2} +2} = \frac{\rho _1}{\rho _2},\\&\frac{(\gamma -1)Ma_{1}^{2} +2}{2\gamma Ma_{1}^{2}-(\gamma -1)} =Ma_{2}^{2} ,\\&{[}2+(\gamma - 1)Ma_{1}^{2} ]\frac{2 \gamma Ma_{1}^{2}-(\gamma -1)}{(\gamma +1)^2Ma_{1}^{2}} = \frac{T_1}{T_2},\\&\frac{1}{\gamma +1} [2 \gamma Ma_{1}^{2}-(\gamma -1) ] = \frac{P_1}{P_2}, \end{aligned} \right. \end{aligned}$$

which relates the physical values before and after the shock. A similar solution strategy is adopted to calculate two accurate and smooth solutions with 8 decimal digits pieced together at the shock as references^32^.

The computational domain is: $x\in \left[ 0,2.25 \right]$. The following boundary conditions are applied in PINNs:

$$\begin{aligned} (\rho _{x=0},T_{x=0},P_{x=0})=(1.0, 1.0, 1.0), \quad P_{x=2.25}=P_{b}. \end{aligned}$$

Therefore, the loss function for the boundary conditions is expressed as MSEs as follows:

13 $$\begin{aligned} MSE_{BC} =MSE_{\rho }+MSE_{T}+MSE_{P} \end{aligned}$$

with:

14 $$\begin{aligned} \begin{aligned}{}&MSE_{\rho } = \frac{1}{N_{BC} } \sum _{j = 1}^{N_{BC}} \left( \left| \rho _{NN}\left( x_{j}=0 \right) -\rho (x_{j}=0)\right| ^{2}\right) ,\\&MSE_{T} = \frac{1}{N_{BC} } \sum _{j = 1}^{N_{BC}} \left( \left| T_{NN}\left( x_{j}=0 \right) -T(x_{j}=0)\right| ^{2}\right) ,\\&MSE_{P} = \frac{1}{N_{BC} } \sum _{j = 1}^{N_{BC}} \left( \left| P_{NN}\left( x_{j}=0 \right) -P(x_{j}=0)\right| ^{2}+\left| P_{NN}\left( x_{j}=2.25 \right) -P(x_{j}=2.25)\right| ^{2}\right) . \end{aligned} \end{aligned}$$

The NN has 3 hidden layers and each layer 30 neurons. $N_{BC}=2$ and $N_F=200$ are evenly distributed in the *x* direction. The training proceeds with Adam optimizer for 2000 epochs and continues with L-BFGS optimizer for 500 epochs. After training, 50 evenly distributed points are selected for predicting solutions.

**Figure 6:**
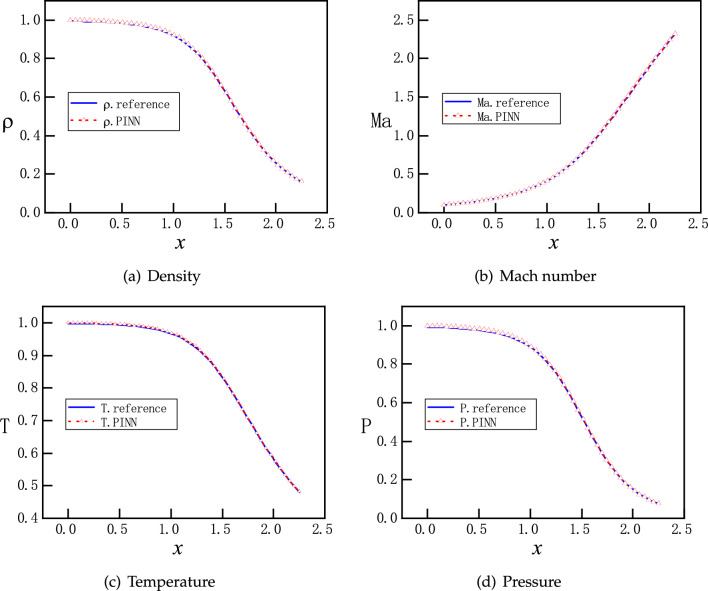
PINNs’ solutions for supersonic continuous flows: with $P_b=0.07726$, the solutions of $\rho$, *Ma*, *T*, and *P* are smooth, with subsonic flow in the converging part while supersonic flow in the diverging part. References are taken from the analytical solutions governed by Eq. (9).

Firstly, $P_b=0.07726$, the flow is subsonic in the converging part and completely supersonic in the diverging part. The solutions from PINNs are shown in the Fig. 6, where the reference solutions according to Eq. (9) are also presented for comparison. We observe that PINNs with a default setting offer excellent accuracy for this type of compressible flow, where there is smooth transition from a subsonic flow to a supersonic flow at the throat $x=1.5$, corresponding to curve F on Fig. 1b.

**Figure 7:**
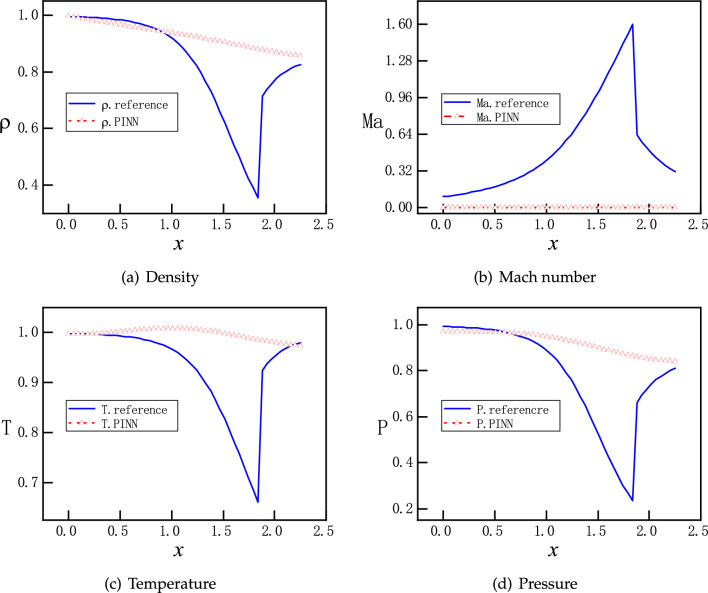
PINNs’ solutions for subsonic and supersonic discontinuous flows. With $P_b=0.81017$, the analytical solutions of $\rho$, *Ma*, *T*, and *P* are governed by by Eqs. (9) and (12). Solutions should be smooth and subsonic in the converging part and become discontinuous at $x=1.875$ in the diverging part, where a normal shock wave is expected. PINNs with default settings fail to reproduce the correct solutions and increasing the number of training points and/or epochs does not help.

**Figure 8:**
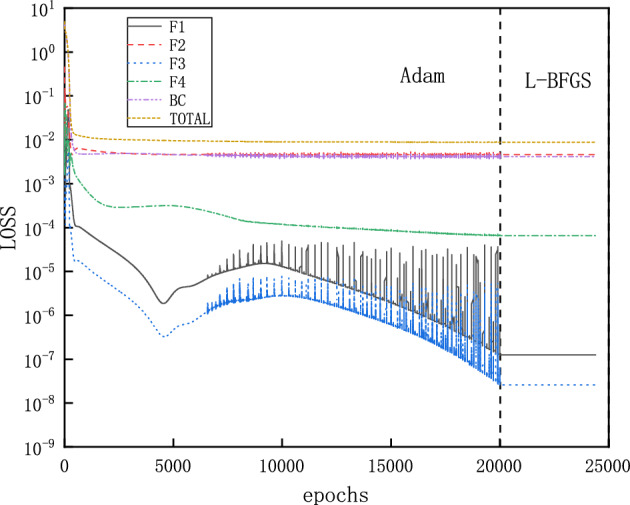
Evolution of the loss function of PINNs for solving the flows in the CD nozzle with $P_b=0.81017$, where a normal shock is expected in the diverging part: with Adam and L-BFGS optimizers, components of the loss function are reluctant to descend towards minimization, especially those for boundary (BC) and momentum equation (F2).

Furthermore, we set $P_{b}=0.81017$. According to Eqs. (9) and (12), a normal shock wave is expected at $x=1.875$ in the diverging part of the nozzle. With a default setting, PINNs offer solutions that are far away from the references, as shown in Fig. 7. An examination at the evolution of the loss function during training reveals that the troublemakers are the losses for the boundary condition and momentum equation, as shown in Fig. 8. Increasing the number of training epochs for ten times from 2500 to 25000 epochs does not improve the descent of the loss function. Enhancing the number of sampling points ten times from 200 to 2000 does not help either (results are not shown).

A few notes are in order. Since we employ PINNs as a direct numerical simulation tool without prior data, they not aware of the shock location in advance and can not distribute more sampling points around the shock. This renders an accurate prediction of the discontinuous flow by PINNs challenging. After a closer inspection on Fig. 7b, we observe that the velocity/Mach number profile is almost flat around zero. This indicates that during the training the optimizer of NNs ignores the residual of momentum equation. Leaving a flat velocity implies all velocity terms can be discarded from $F_1$, $F_2$ and $F_3$ in Eq. (4), since $\partial v/\partial x \approx 0$. We interpret $v(x)=constant$ as a trivial, but wrong solution. For the loss of $F_1$ and $F_3$, the term $\partial A/\partial x$ is known from the geometry, which demands the residuals towards zero, as shown in Fig. 8. However, with the trivial solution of $v(x)=constant$, the pressure cannot satisfy two Dirichlet boundary conditions for the inlet and outlet simultaneously that is , $\partial P/\partial x=0$ is impossible, as shown in Fig. 7d. With $\partial v/\partial x \approx 0$, $\partial P/\partial x$ is the only term left in $F_2$, which does not descend towards zero. Consequently, the losses for boundary condition and $F_2$ are both large and do not descend easily, as shown in Fig. 8.

**Figure 9:**
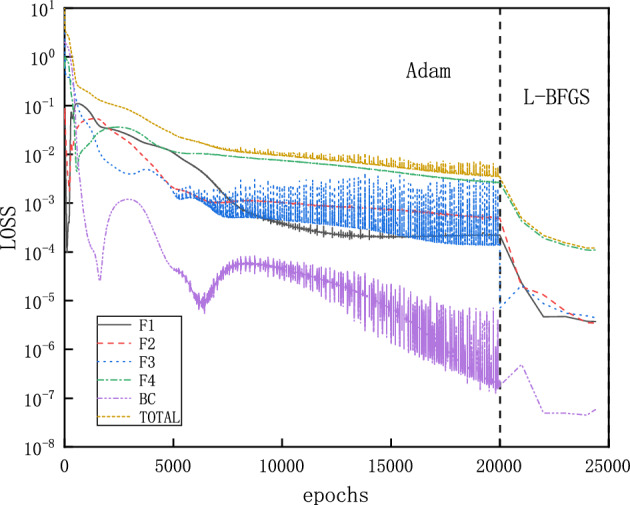
Evolution of the loss function of PINNs for solving the flows in the CD nozzle with $P_b=0.81$, where a normal shock is expected in the diverging part: with Adam and L-BFGS optimizers. With more weight on the momentum equation and hard constrain on the Dirichlet boundary conditions, all components of the loss function descend easily.

**Figure 10:**
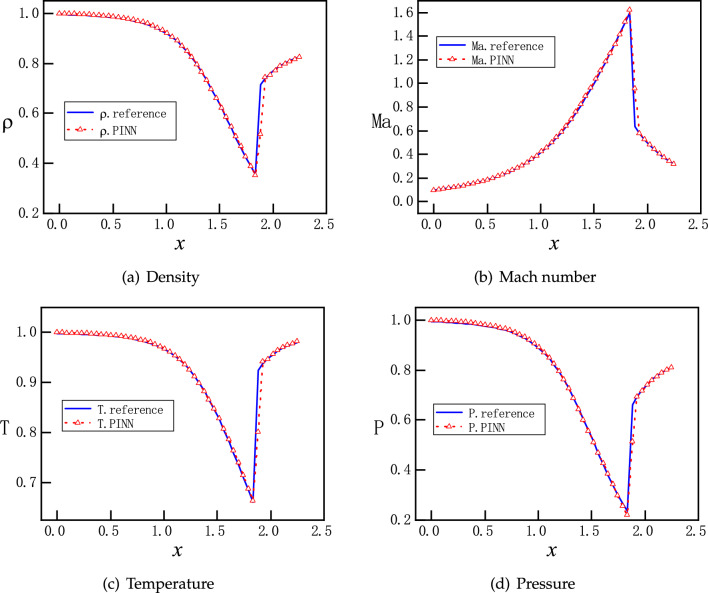
PINNs’ solutions for subsonic and supersonic flows with discontinuity. With $P_b=0.81017$, the analytical solutions of $\rho$, *Ma*, *T*, and *P* are governed by by Eqs. (9) and (12). Solutions should be smooth and subsonic in the converging part and become discontinuous at $x=1.875$ in the diverging part, where a normal shock wave is expected. PINNs with a proper weight on the momentum loss function and hard constraint on the Dirichlet boundary conditions identify shock location and reproduce accurately discontinuous flows at ease.

Motivated by the observations and speculations above, we introduce two small modifications into the vanilla version PINNs. Firstly, we choose to redistribute the weights between the components of the loss function for the PDEs as $\omega _{1}:\omega _{2}:\omega _3:\omega _4=1:20:1:1$, to enhance the descent of momentum residual. In addition, we employ hard constraints of pressure to satisfy the Dirichlet boundary conditions exactly, instead of minimizing the MSEs. Specifically, the values of the pressure are fixed to be 1 and 0.81 at the inlet and outlet, so that the loss about the pressure boundary condition is fixed to be 0 during the training process, instead of being optimized like the other parts of the loss. A more detailed description of the hard constrained boundary conditions can be found^36^. The NN still has 3 hidden layers and each layer has 30 neurons. Moreover, 2000 uniformly distributed points in the x direction are selected as training points. The training proceeds with Adam for 20000 epochs and continues with L-BFGS for 5000 epochs. The upgraded evolution of loss function during training is shown in Fig. 9, where a clear descent for all components is observed. Furthermore, new predictions of PINNs are in Fig. 10, where a normal shock wave is observed at $x=1.875$ and meanwhile sharp profiles are reproduced accurately for $\rho$, *Ma*, *T* and *P* with 50 points. We emphasize that with this new setting, the location of the shock is identified by PINNs automatically without any other efforts. We note that both the modified weights and hard constraints on the Dirichlet boundary conditions are necessary for the accurate solutions.

**Figure 11:**
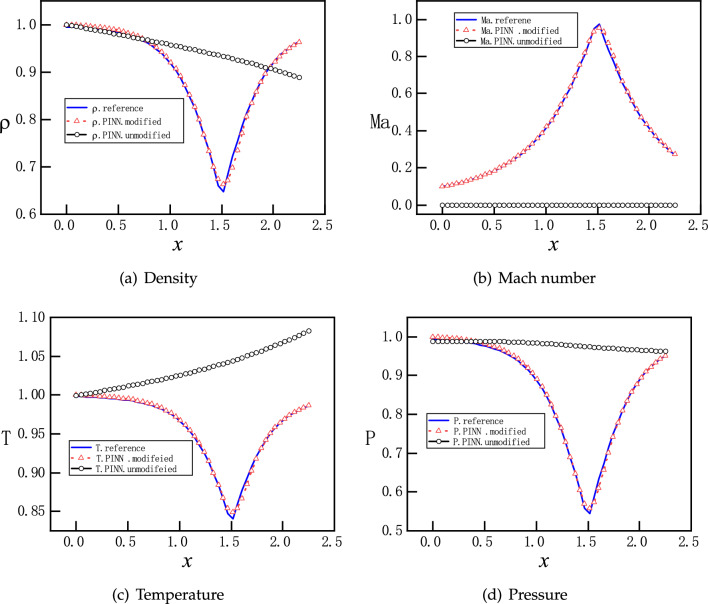
PINNs’ solutions for subsonic flows. For $P_b=0.95055$, a subsonic flow is expected in the entire nozzle as curve B on Fig. 1b. PINNs with a default setting cannot deal with continuous problem properly, whereas PINNs with modified weights and hard constrains on the Dirichlet boundary conditions predict the flows accurately.

Lastly, we set $P_b=0.95055$ so that a subsonic flow is expected in the entire nozzle. Results of two versions of PINNs are presented in Fig. 11. We observe that PINNs with a default setting provides a trivial and wrong solution of $v(x)\approx 0$ and also incorrect solutions for $\rho$, *T* and *P*. With modified weights of on the momentum loss and hard constrains on the Dirichlet boundary conditions, PINNs are able to predict accurately the continuous subsonic flow in the entire nozzle, which turns trend after the throat at $x=1.5$. This corresponds to curve B on Fig. 1b.

With this new setting, PINNs are also able to reproduce Figs. 5 and 6, results of which are omitted. Therefore, we shall continue to use this version of PINNs for the rest of the work.

### Effects of resolution

**Figure 12:**
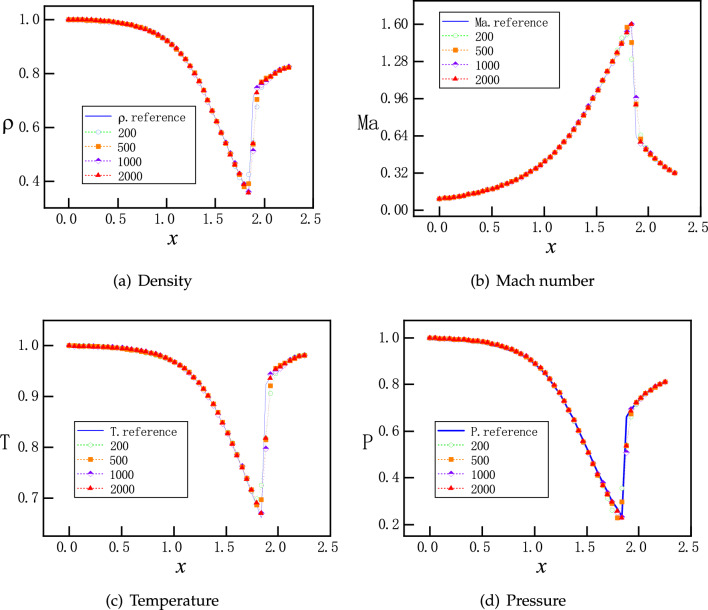
The number of training points is 200,500,1000 and 2000, respectively. The prediction has 50 points. They are all evenly distributed in the *x* direction.

In this section, the effects of the number of training points for PINNs are explored. We exemplify this study by $P_b=0.81017$, where a normal shock is expected in the diverging part of the nozzle. Four different numbers of training points are selected: 200, 500, 1000 and 2000, which are uniformly distributed in the x direction. After training, physical quantities at 50 uniformly distributed points are selected for predictions. As shown in Fig. 12, with all four resolutions of training, results are stable and accurate for density, Mach number, temperature and pressure. It is worth noting that there is no Gibbs phenomenon, which is typically observed in traditional numerical methods.

### An inverse problem

**Figure 13:**
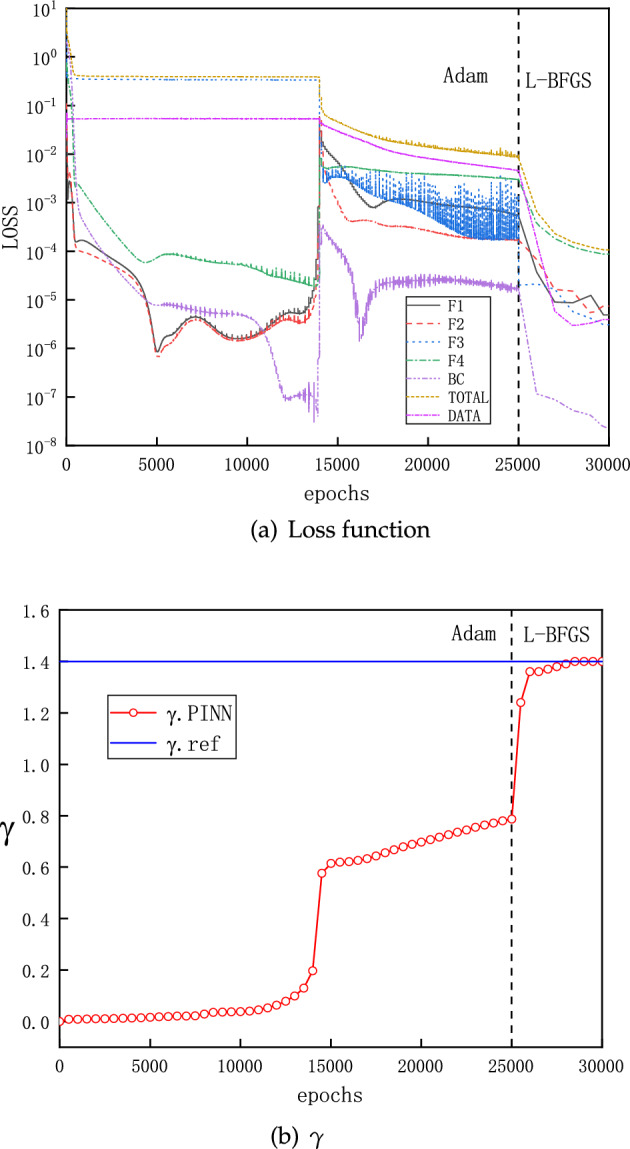
Evolution of sub-loss functions and $\gamma$ as the number of training epochs increases for the inverse problem.

Here we demonstrate the effectiveness of PINNs for an inverse problem. We consider the case of a normal shock taking place in the diverging part of the CD nozzle, which corresponds to the forward problem presented on Fig. 10 with $P_b=0.81017$. We take 50 solution points of pressure uniformly as known and demand PINNs to deliver profiles of $\rho$, *Ma* and *T*, and as well as the specific heat ratio $\gamma$ in the equation of state. On Fig. 13, we present the convergence for each sub-loss function as the number of training epochs increases. Meanwhile, we observe that the prediction of $\gamma$ reaches correctly the target value 1.4 in the end. Results of $\rho$, *v*(*Ma*) and *T* in the inverse problem are indistinguishable from those on Fig. 10 and therefore, are omitted here.

## Time-dependent solutions

A more realistic flow in a CD nozzle has transit states from rest to the steady state. There are no analytical solutions for this time-dependent flow, which makes a numerical procedure necessary. In this section, we leverage the power of PINNs to tackle this time-dependent problem, possibly with normal shocks. In Sect. 4.1, PINNs are employed to solve the time-dependent flows, for which steady state is supersonic in the diverging part of the nozzle. In Sect. 4.2, the influences of NNs’ parameters, such as the size, the number of training points and different distributions of training points on the solution accuracy are discussed. In Sect. 4.3, different initial conditions for the time-dependent flows are investigated, for which steady state has a normal shock.

### Subsonic-supersonic continuous flow

In this section, we solve the unsteady flow in the nozzle for the initial and boundary conditions given as follows

$$\begin{aligned}{} & {} \left( \rho ^{t=0}_{x},v_{x}^{t=0},T_{x}^{t=0},P_{x}^{t=0}\right) =(1.0, 0.0, 1.0, 1.0),\\{} & {} \left( \rho ^{t} _{x=0},T_{x=0}^{t},P_{x=0}^{t}\right) =(1.0, 1.0, 1.0),\quad P_{x=2.25}^{t}=P_{b}=0.07726. \end{aligned}$$

As shown in the previous section, the flow is supersonic in the diverging part of the nozzle for a steady flow. For a time-dependent flow, the computational domain in space and time is $x\in \left[ 0,2.25 \right]$ and $t\in \left[ 0,8 \right]$, respectively. Furthermore, the loss function for ICs expressed as MSEs is defined as

15 $$\begin{aligned} MSE_{IC} =MSE_{\rho }^{IC}+MSE_{v}^{IC}+MSE_{T}^{IC}+MSE_{P}^{IC} \end{aligned}$$

with:

16 $$\begin{aligned} \begin{aligned}{}&MSE_{\rho }^{IC} = \frac{1}{N_{IC} } \sum _{j = 1}^{N_{IC}} \left( \left| \rho _{NN}\left( x_{j},0 \right) -\rho (x_{j},0)\right| ^{2}\right) ,\\&MSE_{v}^{IC} = \frac{1}{N_{IC} } \sum _{j = 1}^{N_{IC}} \left( \left| v_{NN}\left( x_{j},0 \right) -v(x_{j},0)\right| ^{2}\right) ,\\&MSE_{T}^{IC} = \frac{1}{N_{IC} } \sum _{j = 1}^{N_{IC}} \left( \left| T_{NN}\left( x_{j},0 \right) -T(x_{j},0)\right| ^{2}\right) ,\\&MSE_{P}^{IC} = \frac{1}{N_{IC} } \sum _{j = 1}^{N_{IC}} \left( \left| P_{NN}\left( x_{j},0 \right) -P(x_{j},0)\right| ^{2}\right) . \end{aligned} \end{aligned}$$

In addition, the boundary conditions for pressure are implemented as hard constraints as before. The NN has 3 hidden layers and each layer has 30 neurons. Moreover, 100 points are selected for boundary conditions and 150 points for initial conditions. The training starts with Adam optimizer for 10000 epochs and continues with L-BFGS for 15000 epochs.

**Figure 14:**
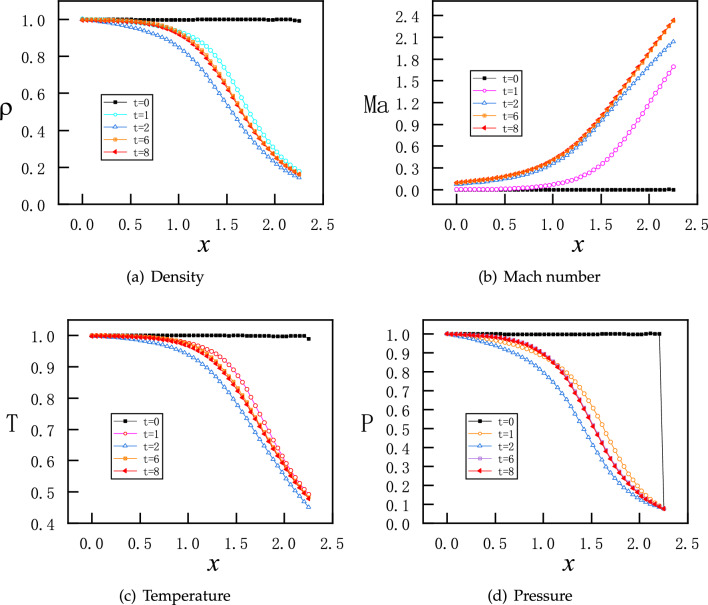
PINNs’ solutions for time-dependent subsonic-supersonic flows at $t=0,1,2,3,6,8$. Density, Mach number, temperature and pressure reach steady states for $t\geqslant 6$.

**Figure 15:**
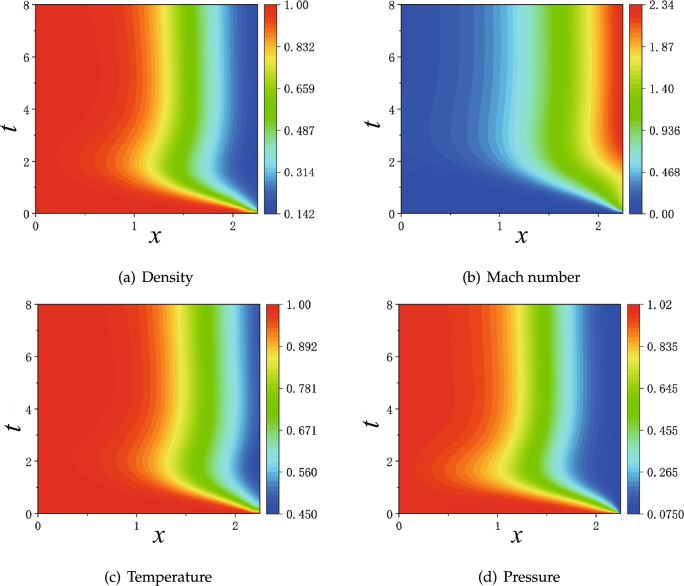
PINNs’s solutions for time-dependent subsonic-supersonic flows with continuous evolutions for density, Mach number, temperature and pressure.

The results of PINNs at 5 discrete time instants are shown in Fig. 14, where the flow quickly reaches supersonic state in the diverging part and become steady state for $t\geqslant 6$. We note that the time evolution for each physical value ($\rho$, *Ma*, *T*, and *P*) is always monotonic along the *x* direction. However, the same evolution of all physical values indicate overshoot in time, that is, steady profiles of $\rho$, *Ma*, *T*, and *P* for $t\geqslant 6$ are in-between profiles at $t=1$ and $t=2$. Furthermore, we present continuous maps for the physical values in both space and time in Fig. 15. Similarly as in the discrete instants in Fig. 14, all four physical values in the continuous color maps are monotonic along the *x* direction, but overshoot in time before reaching steady states.

**Figure 16:**
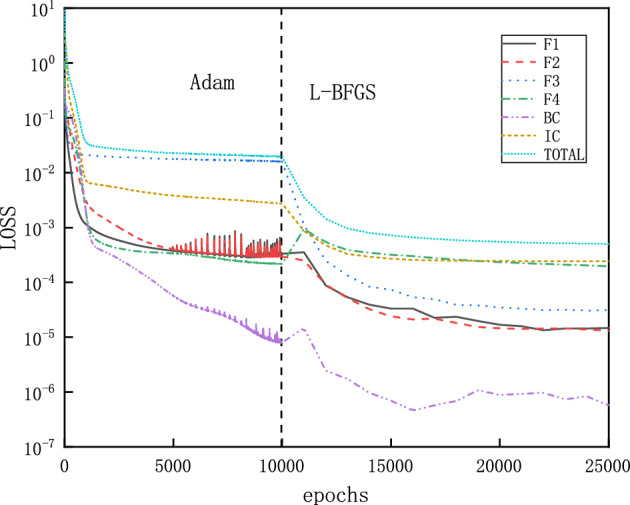
Training loss of PINNs for the continuous time-dependent flows.

The training loss of PINNs for this problem is shown in Fig. 16, where a catenation of L-BFGS after Adam is effective to reduce the loss to a much lower level.

### Exploration of neural networks’ parameters for discontinuous flows

The computational domain is given as $x\in \left[ 0,2.25 \right]$ and $t\in \left[ 0,25 \right]$. The initial and boundary conditions for the time-dependent flow are as follows

$$\begin{aligned}{} & {} \left( \rho ^{t=0}_{x},v_{x}^{t=0},T_{x}^{t=0},P_{x}^{t=0}\right) =(1.0, 0.0, 1.0, 1.0),\\{} & {} \left( \rho ^{t} _{x=0},T_{x=0}^{t},P_{x=0}^{t}\right) =(1.0, 1.0, 1.0), \quad P_{x=2.25}^{t}=P_{b}=0.81017. \end{aligned}$$

The steady state with these boundary conditions was studied in Sect. 3.2, which corresponds to a flow with a normal shock at $x=1.875$ in the diverging part. When solving the unsteady process with discontinuity, we find that the NNs with the previous setting result in a poor prediction for the solutions at steady state. It is understandable, as we have one extra dimension of time for the physical quantities to evolve. Therefore, we commence to explore the effects of the parameters of NNs and examine PINNs’ solutions at steady states after going through the time-dependent states.

**Table 2:** List of four sets of NNs’ parameters. For initial and boundary conditions 150 and 100 points are applied universally. The extra points are within the space-time domain of the diverging part. Corresponding results are shown in Figs. 17 and 18 for $NN_a$ and $NN_d$; Figs. 23, 24 in Appendix A for $NN_b$ and $NN_c$.

Index name	Parameters		
	Layers $\times$ neurons	Regular points	Extra points
${NN_a}$	$3 \times 30$	$100 \times 100$	N.A.
${NN_b}$	$4 \times 50$	$100 \times 100$	N.A.
${NN_c}$	$3 \times 30$	$100 \times 100$	$30 \times 30$
${NN_d}$	$4 \times 50$	$100 \times 100$	$30 \times 30$

We employ four sets of parameters as listed in Table 2, where we have two architectures of NNs: 3 hidden layers $\times$ 30 neurons and 4 hidden layers $\times$ 50 neurons. For the training points, we have $100 \times 100$ regular points uniformly distributed in the whole space-time domain $x \times t \in [0,2.25] \times [0, 25]$. As an attempt to enhance resolution, we add $30 \times 30$ extra points uniformly distributed in the space-time domain of the diverging region of the nozzle $x \times t \in [1.5,2.25] \times [0, 25]$. For the initial and boundary conditions, 150 and 100 points are universally applied, respectively. Each training starts with Adam optimizer for 10000 epochs and continues with L-BFGS for 15000 epochs.

**Figure 17:**
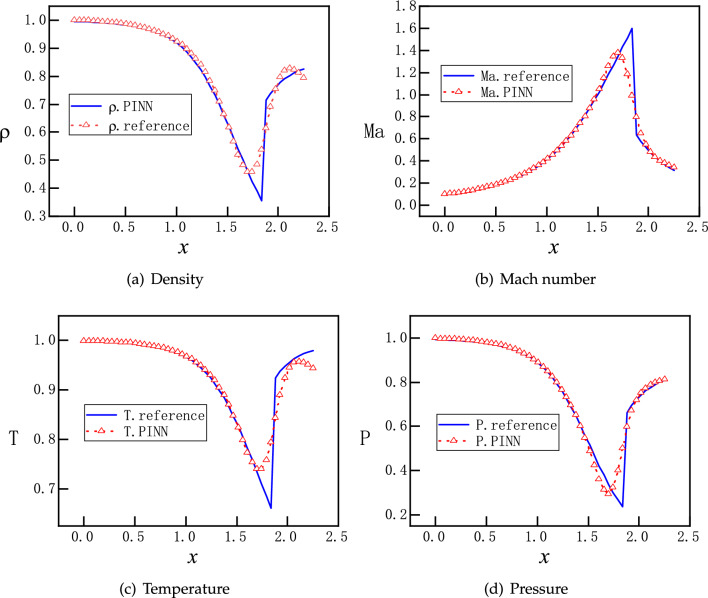
PINNs’ results with setup $NN_a$ for steady states from unsteady process: 3 hidden layers and each layer 30 neurons; Regular $100 \times 100$ training points for space-time domain $x \times t \in [0, 2.25] \times [0, 25].$

**Figure 18:**
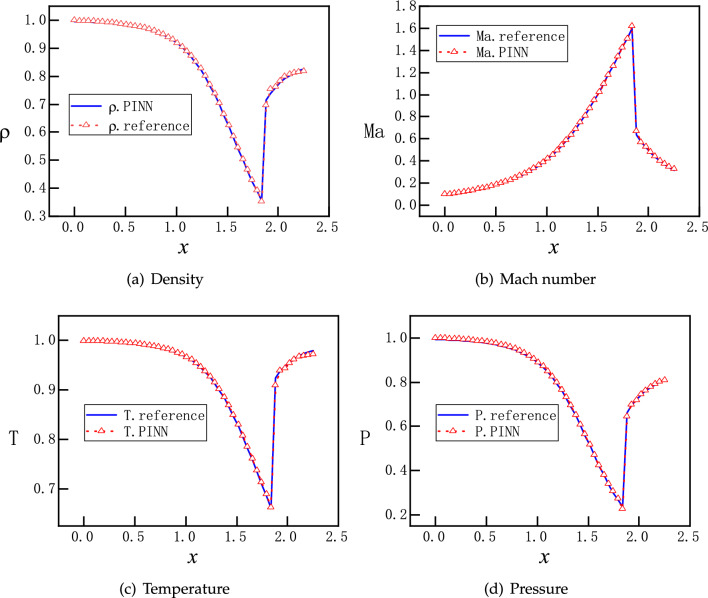
PINNs’ results with setup $NN_d$ for steady states from unsteady process: 4 hidden layers and each layer 50 neurons; Regular $100 \times 100$ training points for space-time domain $x \times t \in [0, 2.25] \times [0, 25].$ Extra $30 \times 30$ training points for space-time domain $x \times t \in [1.5, 2.25] \times [0, 25]$.

For the time being, we discard PINNs’ results at transit states and present solutions at steady states. We observe that the first setup of $NN_a$ reproduces the steady states qualitatively, as shown in Fig. 17, where an norm shock can be identified albeit at a location biased towards upstream. The overall profiles of $\rho$, *Ma*, *T* and *P* are accurate for the subsonic region before the shock, and deteriorate evidently after the shock. Next, we consider two individual improvements over the NNs: one is with enhanced number of layers and neurons corresponding to setup $NN_b$; another is with enlarged number of sampling points in the diverging region of the nozzle corresponding to setup $NN_c$. Both setups improve the results substantially, but the solutions are still not sufficiently accurate, as shown in Figs. 23 and 24 in Appendix A. Furthermore, we look at PINNs’ results with setup $NN_d$ in Fig. 18, which represents the combined efforts of improvement on the NN’s achitecture and increased number of training points. We observe that solutions from PINNs solving an unsteady process predict the steady states correctly, with the same accuracy as the solutions of a steady flow solved by PINNs.

**Figure 19:**
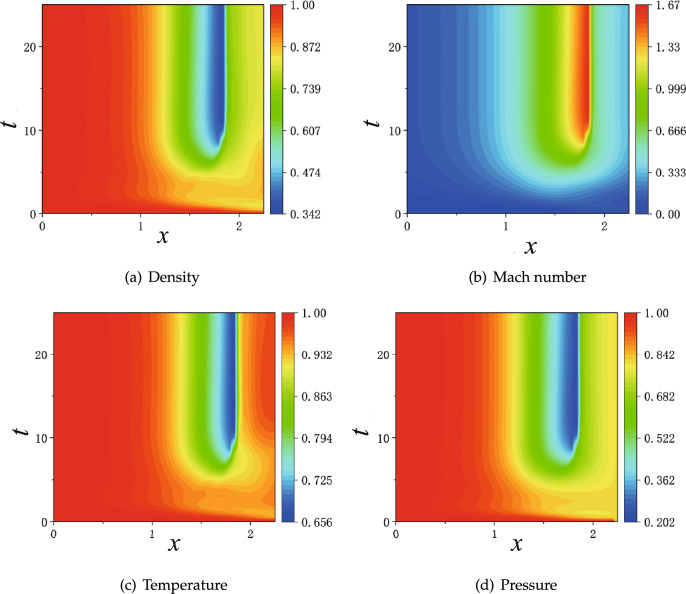
PINNs’s results for time-dependent subsonic-supersonic flows with discontinuous evolutions for density, Mach number, temperature and pressure. The setup of PINNs is with $NN_{d}$ as listed in Table 2.

Lastly, we present the complete evolution of $\rho$, *Ma*, *T* and *P* in Fig. 19. From the color maps of physical values in the whole space-time domain, we may divide approximately the evolution into three stages in time. At the first stage for $t \lesssim 5$, the flow has clearly subsonic characteristics: all four physics values develop rapidly, but always have continuous profiles. At the second stage between $5 \lesssim t \lesssim 8.5$, supersonic characteristics of the flow arise at downstream of the throat at $x=1.5$, but the physical values are still continuous. At the third stage for $t \gtrsim 8.5$, discontinuous phenomena emerge. Later on, physical values settle for $t \gtrsim 10$ , where a sharp interface for physics values is evident at $x=1.875$.

### Two initial conditions for discontinuous flows

The transient flows may be very different under distinct initial conditions. Therefore, we consider the effects due to two initial conditions. The first initial condition as before is repeated as follows

$$\begin{aligned} \left( \rho ^{t=0}_{x},v_{x}^{t=0},T_{x}^{t=0},P_{x}^{t=0}\right) =(1.0, 0.0, 1.0, 1.0). \end{aligned}$$

This corresponds to the scenario when the inlet is already open and the outlet is closed before the flow. Therefore, the density and pressure inside the nozzle are identical as the stagnation values of the inlet. As second initial condition, we consider that the outlet is open and the inlet is closed before the flow. Therefore, the density and pressure inside the nozzle are identical as the values of the outlet. The values for the second initial condition are as follows

$$\begin{aligned} \left( \rho ^{t=0}_{x},v_{x}^{t=0},T_{x}^{t=0},P_{x}^{t=0}\right) =({\rho }_{b}=0.81017, 0.0, 1.0, P_{b}=0.81017). \end{aligned}$$

For both cases, the temperature initially has a stagnation value of $T=1.0$ in the entire nozzle and the boundary conditions are identical as follows

$$\begin{aligned} \left( \rho ^{t} _{x=0},T_{x=0}^{t},P_{x=0}^{t}\right) =(1.0, 1.0, 1.0), \quad P_{x=2.25}^{t}=P_{b}=0.81017. \end{aligned}$$

**Figure 20:**
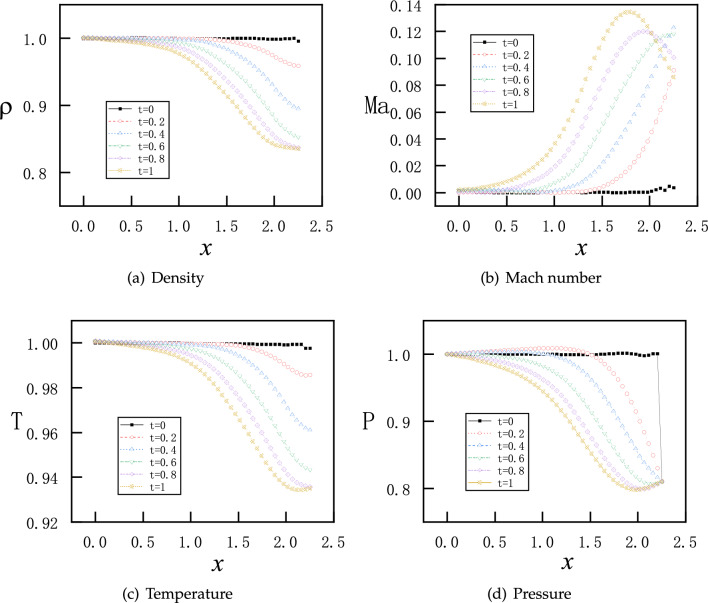
PINNs’ results at 6 instants of short time for time-dependent discontinuous flows with the first initial conditions: before the flow the inlet is open while the outlet is closed.

**Figure 21:**
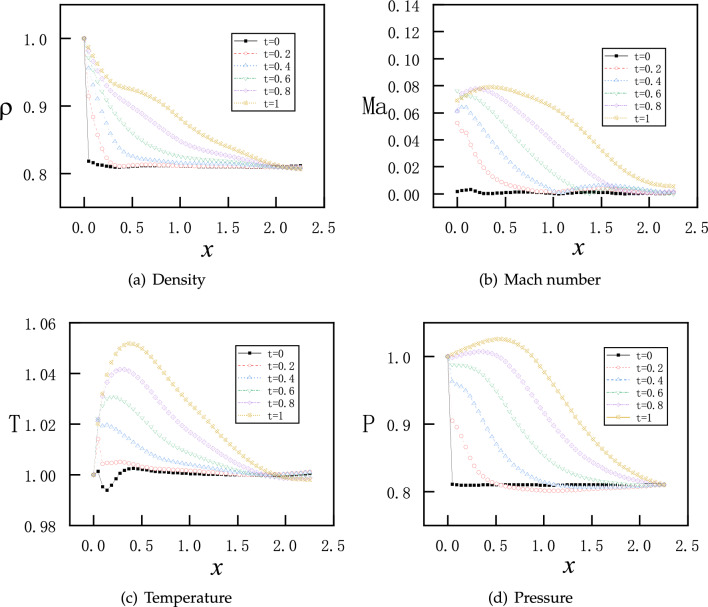
PINNs’ results at 6 instants of short time for time-dependent discontinuous flows with the second initial conditions: before the flow the inlet is closed while the outlet is open.

We observe that both flows develop rapidly from the two different initial conditions. After $t=1$, the two paths of transition states of all physical values are already very similar, as shown in Figs. 25 and 26 in Appendix B. Therefore, here we only present the two sets of results for $t \in [0, 1]$ in Figs. 20 and 21. With the first initial conditions, $\rho$, *v* and *T* evolve quickly but smoothly except for *P*, as there is a discontinuous jump between the boundary value and inner initial values at the outlet. Nevertheless, the pressure becomes continuous after $t=0.2$, as shown in Fig. 20d. With the second initial conditions, *v* and *T* values evolve quickly but smoothly except for $\rho$ and *P*, as there are discontinuous jumps between the boundary value and inner initial values at the inlet. Nevertheless, both the density and pressure become continuous after $t=0.2$, as shown in Fig. 21a,d.

## Solutions in conservative form

In classical numerical methods a conservative form of the PDEs is in favor and numerical solutions of the non-conservative form tend to be unstable. Therefore, we consider the solution procedure of PINNs in the context of conservative form, which is given as follows

17 $$\begin{aligned} \partial _t U + \nabla \cdot K = J. \end{aligned}$$

*U*, *K* and *J* are vectors: $$U=\begin{pmatrix} U_1 \\ U_2\\ U_3 \end{pmatrix},$$ $$K=\begin{pmatrix} K_1 \\ K_2\\ K_3 \end{pmatrix}$$ $$J=\begin{pmatrix} J_1 \\ J_2\\ J_3 \end{pmatrix}$$, and they are defined as

18 $$\begin{aligned}{}&\left\{ \begin{aligned}{}&\rho A = U_1,\\&\rho Av = U_2,\\&\rho A\left( \frac{T}{\gamma -1} +\frac{\gamma }{2} v^{2} \right) = U_3. \end{aligned} \right. \end{aligned}$$

19 $$\begin{aligned}{}&\left\{ \begin{aligned}{}&\rho Av = K_1,\\&\rho Av^2 + \frac{1}{\gamma } PA= K_2,\\&\rho A\left( \frac{T}{\gamma -1} +\frac{\gamma }{2} v^{2} \right) +PAv = K_3. \end{aligned} \right. \end{aligned}$$

20 $$\begin{aligned}{}&\left\{ \begin{aligned}{}&0 = J_1,\\&\frac{1}{\gamma } P\frac{\partial A}{\partial x} = J_2,\\&0 = J_3. \end{aligned} \right. \end{aligned}$$

**Figure 22:**
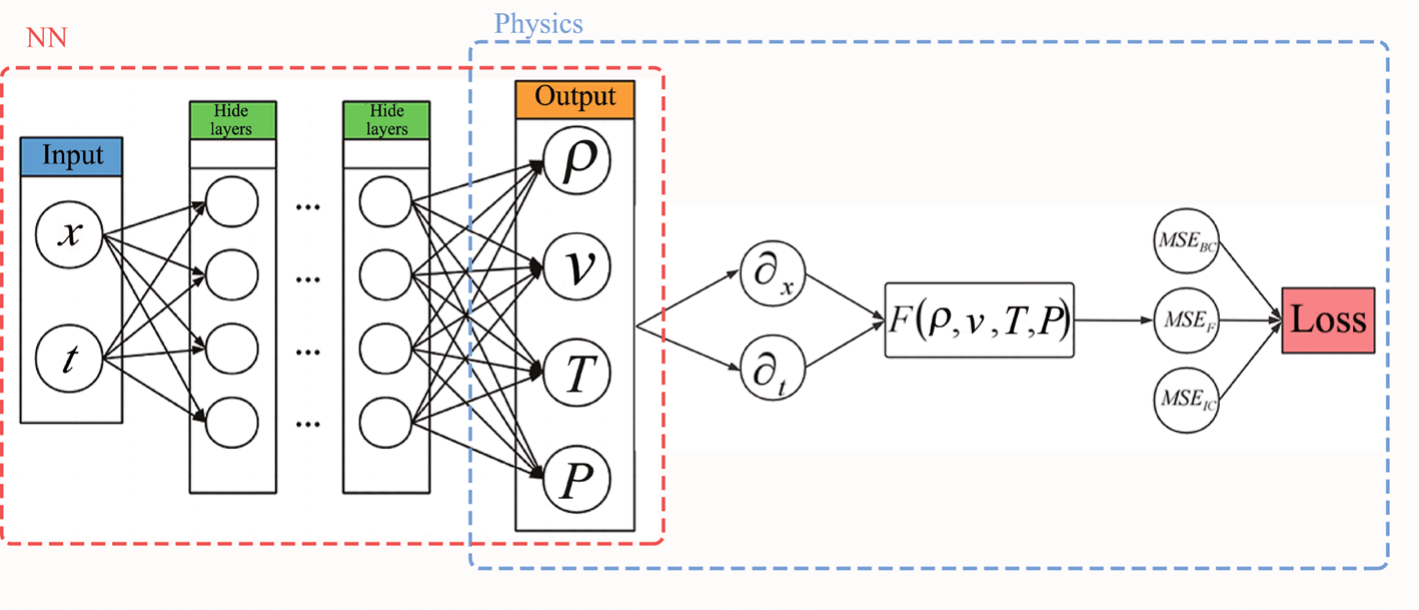
The structure of PINNs for Euler equations in conservative form.

Accordingly, we have to adjust the outputs of the NNs to be $$U_1,U_2,U_3 $$ and *P*, as shown in Fig. 22. To facilitate the construction of loss function, we have to rewrite $$K_i$$ as a function of the NNs’ outputs, namely, $$U_i$$, as follows

21 $$\begin{aligned} \left\{ \begin{aligned}{}&U_2 = K_1,\\&\frac{U_{2}^{2} }{U_1} +\frac{\gamma -1}{\gamma } \left( U_3-\frac{\gamma }{2}\frac{U_{2}^{2} }{U_1} \right) = K_2,\\&\gamma \frac{U_{2}U_{3}}{U_1} -\frac{\gamma (\gamma -1)}{2} \frac{U_{2}^{3} }{U_{1}^{2} } = K_3, \end{aligned} \right. \end{aligned}$$

where we have used the fact $P=\rho T$ from the equation of state. Therefore, each component of the loss function is as follows

22 $$\begin{aligned} \left\{ \begin{aligned}{}&\frac{\partial U_{1} }{\partial t} +\frac{\partial K_{1} }{\partial x} =F_1(x,t),\\&\frac{\partial U_{2} }{\partial t} +\frac{\partial K_{2} }{\partial x}-\frac{1}{\gamma }P\frac{\partial A}{\partial x} =F_2(x,t),\\&\frac{\partial U_{3} }{\partial t} +\frac{\partial K_{3} }{\partial x} =F_3(x,t),\\&P-\frac{U_{1} }{A} \left( \gamma -1 \right) \left( \frac{U_3}{U_1}-\frac{\gamma }{2} \frac{U_{2}^{2} }{U_{1}^{2} } \right) =F_4(x,t). \end{aligned} \right. \end{aligned}$$

Here $F_1(x,t)$, $F_2(x,t)$, $F_3(x,t)$, and $F_4(x,t)$ represent residuals of the mass, momentum, energy, and state equations, respectively. For automatic differentiation, we have to expand all terms $K_i$ in Eq. (22) with $U_i$ from Eq. (21). These expansions quickly become unpleasant with many dividing operations, which poses challenges for gradient calculations and optimizations of the NNs’ parameters. Despite our best efforts, the loss function does not descend easily and no meaningful predictions are made by PINNs with the conservative form. Switching from Adam to L-BFGS optimizer does not help and the loss remains at the level well above $10^{-4}$, and therefore the outputs of PINNs are physically meaningless and are not shown here.

## Conclusions

We applied physics-informed neural networks or PINNs to directly solve steady and time-dependent compressible flows within a converging–diverging channel, corresponding to an unsupervised learning. With different boundary conditions, the flow may be completely subsonic, subsonic via a smooth transition to supersonic, or further from supersonic via a discontinuous transition back to subsonic.

Firstly, for a simple diverging channel, when sonic boundary conditions are imposed at the inlet and the outlet are left intentionally free, PINNs provide a subsonic or a supersonic continuous solution randomly and deliberately avoid solutions with discontinuity. The stochastic outcomes result from the randomness during initialization of the neural networks (NNs); Secondly, for a converging–diverging nozzle, PINNs with a default setting cannot capture transition from supersonic to subsonic flows with a discontinuity for the normal shock, although given proper boundary conditions at both the inlet and outlet. Instead, the loss function is unwilling to descend during training and consequently, a trivial solution with zero velocity and incorrect continuous profiles for other physical values are obtained. The two examples above indicate that during training the optimizer somehow “minimizes its efforts” before minimizing the loss function: it is inclined to offer smooth solutions (right or wrong) and reluctant to find discontinuous solutions. Hence, a small remedy of PINNs is pertinent.

After a close inspection on the descent of each component of the loss function and predictions of the physical values, we promote to put 20 times more weight on the minimization of the momentum equation and meanwhile enforce hard constraints on the boundary conditions of pressure. This exertion is coincident with a recent effort put forward by Perdikaris’ group^37^, where a dynamic weight is proposed to balance the gradients among different components of the loss function, to mitigate gradient vanishing. For the problems considered in this work, we acknowledge a constant heavy weight for the momentum loss being adequate for accurate solutions, after a couple of numerical experiments with trial and error.

With 90 neurons and 100 training points, the so-modified version of PINNs is able to deliver accurate solutions at steady states for subsonic flows, supersonic flows and mixture of both with a norm shock as sharp transition from supersonic to subsonic flows. For unsteady processes, with 200 neurons and 16900 training points, PINNs are able to predict accurately the time-dependent flows until steady states. Whether for steady or unsteady flows, PINNs are able to identify the locations of shocks accurately and offer very sharp profiles for the transitions. In low-order CFD methods, one may need to refine the grid points after the sharp transition is located. In high-order CFD methods, one may encounter the the Gibbs phenomenon near the transition. Following an optimization routine, PINNs avoid the above two drawbacks. These results are promising, as they encourage us to apply PINNs to solve more discontinuous physics phenomena and to replace/supplement traditional numerical schemes to a certain extent. Finally, when the PDEs are expressed in the conservative form, which is in favor by traditional numerical schemes, the output terms of the NNs and their corresponding loss function are entangled and not viable for an effective optimization. Consequently, the predictions offered by PINNs are incorrect. This indicates that PINNs prefer the simple differential form of the PDEs over the conservative form, as the former is more appropriate for straightforward automatic differentiation during optimization.

We envisage two research lines beyond this work. One is to explore PINNs as a direct numerical solver for more general compressible flows in two and three dimensions, especially with shock phenomena. From the performance on one-dimensional flows, it seems promising for PINNs to solve more compressible/discontinuous flows, where no exquisite shock-capturing schemes are essential. Another one is to supply PINNs with partially available experimental data, such as a few pressure values via sensors within the flow, to recover other physics values and/or to estimate unknown coefficients in the PDEs, such as wall frictions.

The code used in this manuscript can be found at the following link. https://github.com/szl-c/pinn_CDnozzle

### Supplementary Information

Supplementary Figures.

## Data Availability

The data that support the findings of this study are available from the corresponding author upon reasonable request.

## References

[1] White, Frank M. *Fluid mechanics* Vol. 8 (McGraw Hill, 2016).

[2] Dafermos, Constantine M. *Hyperbolic conservation laws in continuum physics* Vol. 3 (Springer, 2005).

[3] Anderson, John D. *Computational fluid dynamics* (McGraw-Hill, Inc., 1995).

[4] LeVeque, Randall J. *Finite volume methods for hyperbolic problems* Vol. 31 (Cambridge university press, 2002).

[5] Shu, C.-W. A brief survey on discontinuous Galerkin methods in computational fluid dynamics. *Adv. Mech.* **43**(6), 541–553 (2013).

[6] Jiang, G.-S. & Shu, C.-W. Efficient implementation of weighted ENO schemes. *J. Comput. Phys.* **126**(1), 202–228 (1996). 10.1006/jcph.1996.0130

[7] Wang, Z. J. High-order methods for the Euler and Navier-stokes equations on unstructured grids. *Prog. Aerosp. Sci.* **43**(1–3), 1–41 (2007). 10.1016/j.paerosci.2007.05.001

[8] Jiang, L., Jie, W., Yang, L. & Dong, H. Gas kinetic flux solver based finite volume weighted essentially non-oscillatory scheme for inviscid compressible flows. *Appl. Math. Mech.* **44**(6), 961–980 (2023). 10.1007/s10483-023-3009-9

[9] Jian, Yu., Yan, C. & Jiang, Z. Revisit of dilation-based shock capturing for discontinuous Galerkin methods. *Appl. Math. Mech.* **39**, 379–394 (2018). 10.1007/s10483-018-2302-7

[10] Hou, Y., Jin, K., Feng, Y. & Zheng, X. High-order targeted essentially non-oscillatory scheme for two-fluid plasma model. *Appl. Math. Mech.* **44**(6), 941–960 (2023). 10.1007/s10483-023-3003-6

[11] Michoski, Craig, Milosavljevic, Milos, Oliver, Todd, & Hatch, David. Solving irregular and data-enriched differential equations using deep neural networks. *arXiv preprint* arXiv:1905.04351 (2019).

[12] Wang, B., Wang, Q., Zhou, Q. & Liu, Y. Active control of flow past an elliptic cylinder using an artificial neural network trained by deep reinforcement learning. *Appl. Math. Mech.* **43**(12), 1921–1934 (2022). 10.1007/s10483-022-2940-9

[13] Bezgin, D. A., Schmidt, S. J. & Adams, N. A. Weno3-nn: A maximum-order three-point data-driven weighted essentially non-oscillatory scheme. *J. Comput. Phys.* **452**, 110920 (2022). 10.1016/j.jcp.2021.110920

[14] Liu, Z., Yang, Y. & Cai, Q. Neural network as a function approximator and its application in solving differential equations. *Appl. Math. Mech.* **40**(2), 237–248 (2019). 10.1007/s10483-019-2429-8

[15] Magiera, J., Ray, D., Hesthaven, J. S. & Rohde, C. Constraint-aware neural networks for riemann problems. *J. Comput. Phys.* **409**, 109345 (2020). 10.1016/j.jcp.2020.109345

[16] Schwander, L., Ray, D. & Hesthaven, J. S. Controlling oscillations in spectral methods by local artificial viscosity governed by neural networks. *J. Comput. Phys.* **431**, 110144 (2021). 10.1016/j.jcp.2021.110144

[17] Bezgin, D. A., Schmidt, S. J. & Adams, N. A. A data-driven physics-informed finite-volume scheme for nonclassical undercompressive shocks. *J. Comput. Phys.* **437**, 110324 (2021). 10.1016/j.jcp.2021.110324

[18] Raissi, M., Perdikaris, P. & Karniadakis, G. E. Physics-informed neural networks: A deep learning framework for solving forward and inverse problems involving nonlinear partial differential equations. *J. Comput. Phys.* **378**, 686–707 (2019). 10.1016/j.jcp.2018.10.045

[19] Karniadakis, G. E. *et al.* Physics-informed machine learning. *Nat. Rev. Phys.* **3**(6), 422–40 (2021). 10.1038/s42254-021-00314-5

[20] Jeremy, Yu., Lu, L., Meng, X. & Karniadakis, G. E. Gradient-enhanced physics-informed neural networks for forward and inverse PDE problems. *Comput. Methods Appl. Mech. Eng.* **393**, 114823 (2022). 10.1016/j.cma.2022.114823

[21] Wang, S., Xinling, Yu. & Perdikaris, P. When and why Pinns fail to train: A neural tangent kernel perspective. *J. Comput. Phys.* **449**, 110768 (2022). 10.1016/j.jcp.2021.110768

[22] Mattey, R. & Ghosh, S. A novel sequential method to train physics informed neural networks for Allen Cahn and Cahn Hilliard equations. *Comput. Methods Appl. Mech. Eng.* **390**, 114474 (2022). 10.1016/j.cma.2021.114474

[23] Wang, Sifan, Sankaran, Shyam, & Perdikaris, Paris. Respecting causality is all you need for training physics-informed neural networks. *arXiv preprint* arXiv:2203.07404 (2022).

[24] Wight, C.L. & Zhao, J. Solving Allen-Cahn and Cahn-Hilliard equations using the adaptive physics informed neural networks. *Global Science Press*, (3) (2021).

[25] Xiong, Fansheng, Liu, Li, Liu, Shengping, Wang, Han, & Yong, Heng. Gradient-weighted physics-informed neural networks for one-dimensional euler equation (2022).

[26] Mao, Z., Jagtap, A. D. & Karniadakis, G. E. Physics-informed neural networks for high-speed flows. *Comput. Methods Appl. Mech. Eng.* **360**, 112789 (2020). 10.1016/j.cma.2019.112789

[27] Jagtap, A. D., Kharazmi, E. & Karniadakis, G. E. Conservative physics-informed neural networks on discrete domains for conservation laws: Applications to forward and inverse problems. *Comput. Methods Appl. Mech. Eng.* **365**, 113028 (2020). 10.1016/j.cma.2020.113028

[28] Patel, R. G. *et al.* Thermodynamically consistent physics-informed neural networks for hyperbolic systems. *J. Comput. Phys.* **449**, 110754 (2022). 10.1016/j.jcp.2021.110754

[29] Lou, Q., Meng, X. & Karniadakis, G. E. Physics-informed neural networks for solving forward and inverse flow problems via the Boltzmann-BGK formulation. *J. Comput. Phys.* **447**, 110676 (2021). 10.1016/j.jcp.2021.110676

[30] Zhang, L., Ma, W., Lou, Q., & Zhang, J. Simulation of rarefied gas flows using physics-informed neural network combined with discrete velocity method. Phys. Fluids, 1;35(7) (2023)

[31] Goodfellow, Ian, Bengio, Yoshua & Courville, Aaron. *Deep learning* (The MIT Press, 2016).

[32] www.dept.aoe.vt.edu/ devenpor/aoe3114/calc.html.

[33] Glorot, X., & Bengio, Y. Understanding the difficulty of training deep feedforward neural networks. In *Proceedings of the thirteenth international conference on artificial intelligence and statistics*, pp. 249–256. JMLR Workshop and Conference Proceedings (2010).

[34] Kingma, D.P., & Adam, J.B. A method for stochastic optimization. *arXiv preprint*arXiv:1412.6980 (2014).

[35] Zhu, C., Byrd, R. H., Peihuang, L. & Nocedal, J. Algorithm 778: L-bfgs-b: Fortran subroutines for large-scale bound-constrained optimization. *ACM Trans. Math. Softw. TOMS* **23**(4), 550–560 (1997). 10.1145/279232.279236

[36] Lu, L. *et al.* Physics-informed neural networks with hard constraints for inverse design. *SIAM J. Sci. Comput.* **43**(6), B1105–B1132 (2021). 10.1137/21M1397908

[37] Wang, S., Teng, Y. & Perdikaris, P. Understanding and mitigating gradient flow pathologies in physics-informed neural networks. *SIAM J. Sci. Comput.* **43**(5), A3055-3081 (2021). 10.1137/20M1318043

