# Supplementary material for: Continuous and discontinuous compressible flows in a converging–diverging channel solved by physics-informed neural networks without exogenous data
Source: Sci Rep. 2024 Feb 15;14:3822. doi: 10.1038/s41598-024-53680-2 (PMC11269604; doi:10.1038/s41598-024-53680-2)
Supplement: Supplementary file 1 — Supplementary Figures. [file 41598_2024_53680_MOESM1_ESM.pdf]

Appendix

A Different neural networks' parameters for discontinuous flows

Figs. 23 and 24 present the results of the NNs under the two settings of NN_b and NN_c in Table 2 of Sec. 4.2. Either increasing the number of freedoms of NNs or adding more training points improve predictions to a certain extent, but the results are not sufficiently accurate. More specifically, there are still mismatches for physics values near the shock location from both NN_b and NN_c and profiles of ρ and T from NN_b clearly misalign with analytical references after the shock.

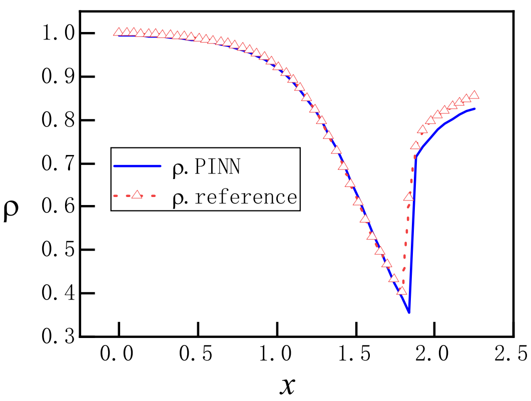

(a) Density

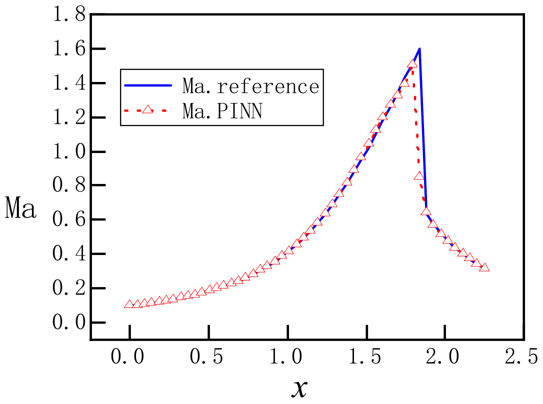

(b) Mach number

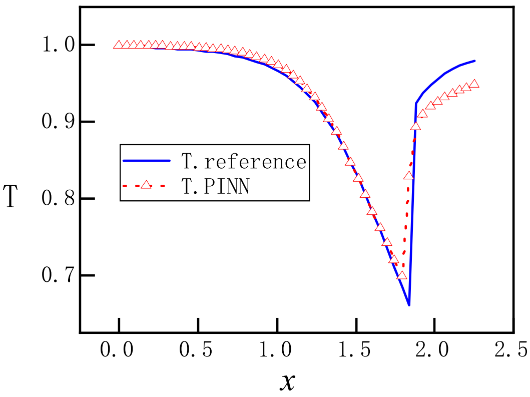

(c) Temperature

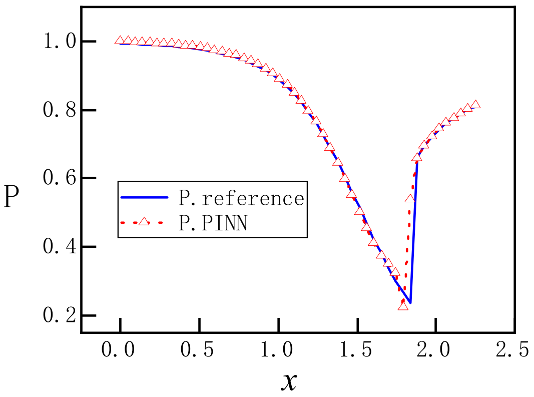

(d) Pressure

Figure 23: PINNs' results with setup NN_b for steady states from unsteady process: 4 hidden layers and each layer 50 neurons; Regular 100×100 training points for space-time domain $x \times t \in [0,2.25] \times [0,25]$.

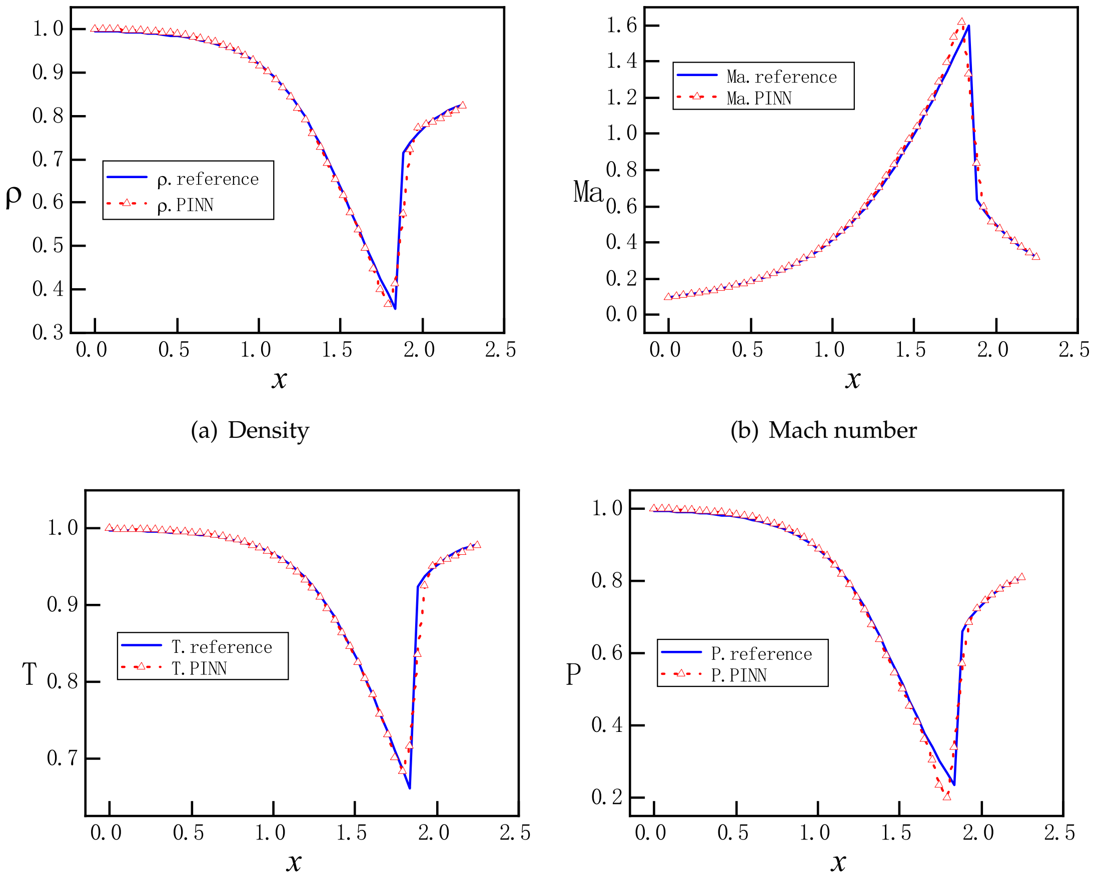

(a) Density

(b) Mach number

(c) Temperature

(d) Pressure

Figure 24: PINNs' results with setup NN_c for steady states from unsteady process: 3 hidden layers and each layer 30 neurons; Regular 100×100 training points for space-time domain $x\times t\in[0,2.25]\times[0,25]$. Extra 30×30 training points for space-time domain $x\times t\in[1.5,2.25]\times[0,25]$.

B Two initial conditions for time-dependent discontinuous flows

The following two diagrams present flows from time 0 to 25 with two different initial conditions in Sec. 4.2. As observed from the two sets of plots, flows do not differentiate from each other after $t=1$ between the two initial conditions. These results suggest that one should examine the flows for $t \in [0,1]$ to compare the differences.

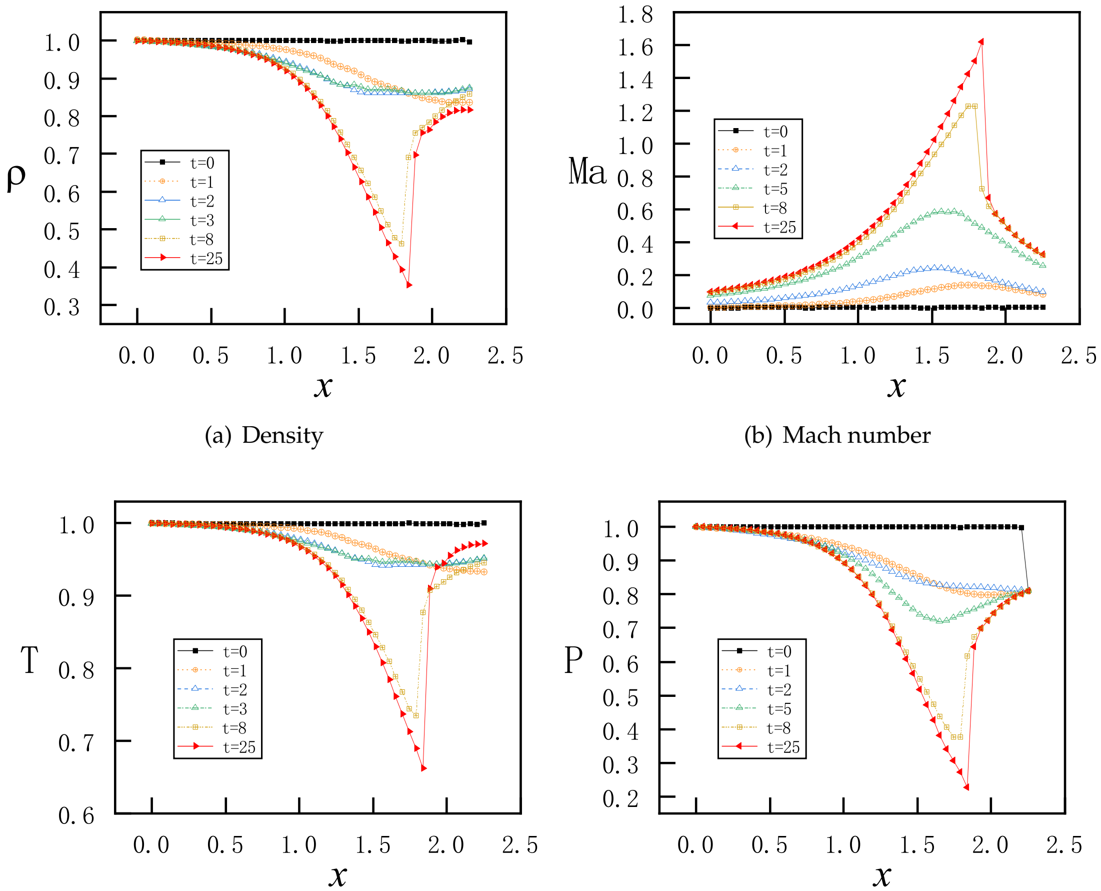

(a) Density (b) Mach number

(c) Temperature (d) Pressure

Figure 25: PINNs' results at 6 instants of long time for time-dependent discontinuous flows with the first initial conditions: before the flow the inlet is open while the outlet is closed.

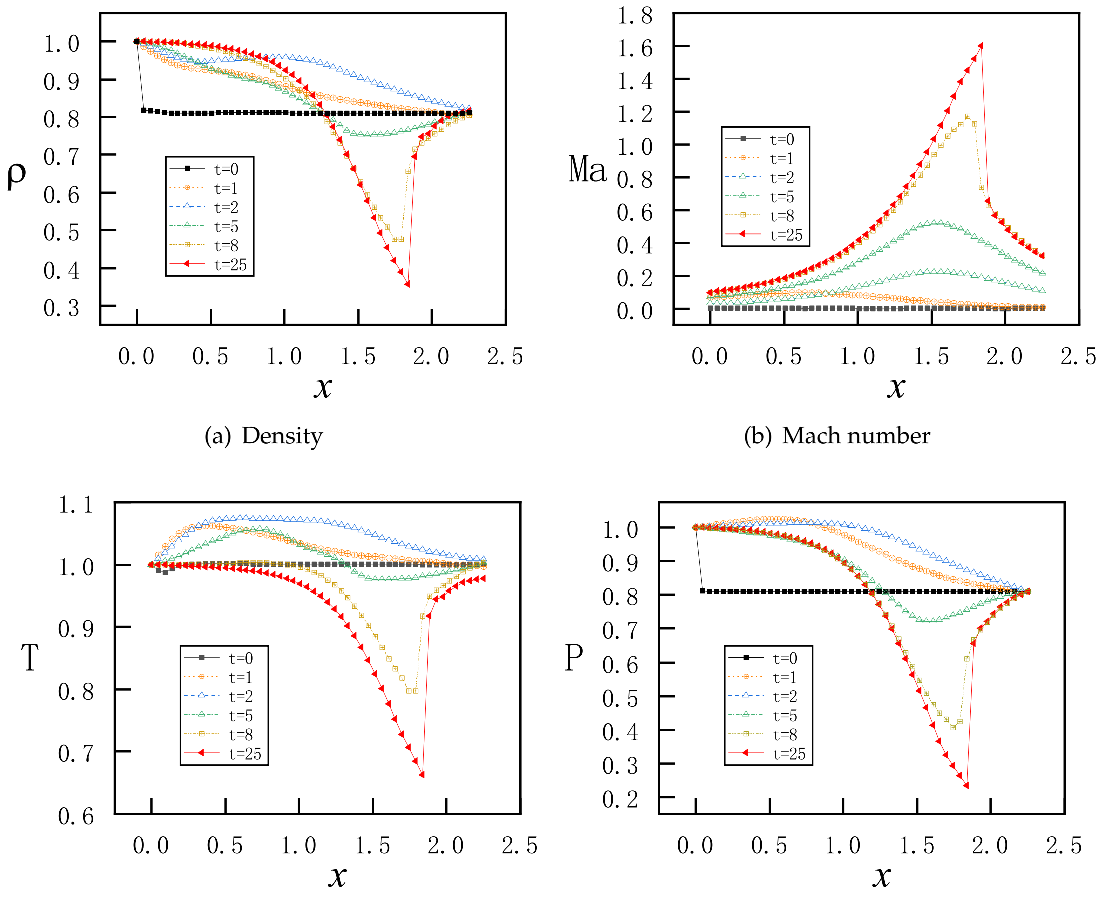

(a) Density (b) Mach number

(c) Temperature (d) Pressure

Figure 26: PINNs' results at 6 instants of long time for time-dependent discontinuous flows with the second initial conditions: before the flow the inlet is closed while the outlet is open.

C The difference between the predicted results of PINNs and the analytical solutions for the discontinuous flow at steady state.

In the typical case of converging-diverging nozzle with shock waves, Fig 10 shows the comparison between PINNs' predicted results and analytic solutions. To show the errors near the shock profile more clearly, absolute values of the differences are taken and shown in logarithmic scale in Fig 27. At locations away from the shock wave ($x = 1.875$), the error between PINNs' prediction result and the analytic solution is stable at a small value, and the error fluctuates significantly near the position close to the shock wave.

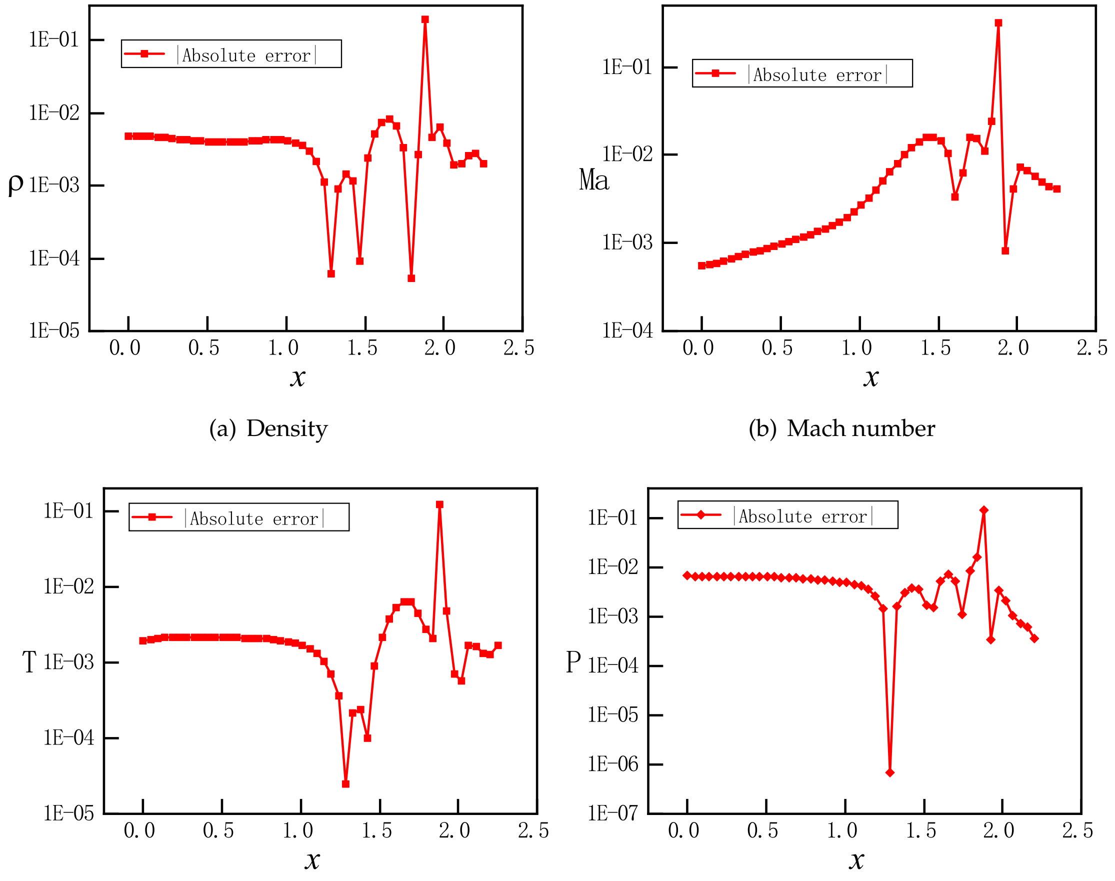

(a) Density (b) Mach number

(c) Temperature (d) Pressure

Figure 27: The absolute difference between the predicted results of PINNs and the analytical solutions in logarithmic scale.
